# Root *NRT*, *NiR*, *AMT*, *GS*, *GOGAT* and *GDH *expression levels reveal NO and ABA mediated drought tolerance in *Brassica juncea* L.

**DOI:** 10.1038/s41598-021-86401-0

**Published:** 2021-04-12

**Authors:** Seema Sahay, Luis Robledo-Arratia, Katarzyna Glowacka, Meetu Gupta

**Affiliations:** 1 Ecotoxicogenomics Lab, Department of Biotechnology, Jamia Millia Islamia, New Delhi, 110025 India; 2 Departamento de Biología, Instituto Nacional de Investigaciones Nucleares (ININ), Ocoyoacac, C.P. 52750 México; 3 Department of Biochemistry and Center for Plant Science Innovation, University of Nebraska-Lincoln, Lincoln, NE 68588 USA

**Keywords:** Biotechnology, Physiology, Plant sciences

## Abstract

Little is known about the interactive effects of exogenous nitric oxide (NO) and abscisic acid (ABA) on nitrogen (N) metabolism and related changes at molecular and biochemical levels under drought stress. The present study highlights the independent and combined effect of NO and ABA (grouped as “nitrate agonists”) on expression profiles of representative key genes known to be involved in N-uptake and assimilation, together with proline metabolism, N–NO metabolism enzyme’s activity and nutrient content in polyethylene glycol (PEG) treated roots of Indian mustard (*B. juncea cv.* Varuna). Here we report that PEG mediated drought stress negatively inhibited growth performance, as manifested by reduced biomass (fresh and dry weight) production. Total N content and other nitrogenous compounds (NO_3_^−^, NO_2_^−^) were decreased; however, NH_4_^+^, NH_4_^+^/ NO_3_^−^ ratio and total free amino acids content were increased. These results were positively correlated with the PEG induced changes in expression of genes and enzymes involved in N-uptake and assimilation. Also, PEG supply lowered the content of macro- and micro-nutrients but proline level and the activity of ∆^1^-pyrroline-5-carboxylate synthetase increased indicating increased oxidative stress. However, all these responses were reversed upon the exogenous application of nitrate agonists (PEG + NO, PEG + NO + ABA, and PEG + ABA) where NO containing nitrate agonist treatment i.e. PEG + NO was significantly more effective than PEG + ABA in alleviating drought stress. Further, increases in activities of L-arginine dependent NOS-like enzyme and S-nitrosoglutathione reductase were observed under nitrate agonist treatments. This indicates that the balanced endogenous change in NO and ABA levels together during synthesis and degradation of NO mitigated the oxidative stress in Indian mustard seedlings. Overall, our results reveal that NO independently or together with ABA may contribute to improved crop growth and productivity under drought stress.

## Introduction

Drought stress is the most dominant abiotic stress that severely affects plant growth performance and productivity, particularly in arid and semi-arid areas all around the world^1,2^. Drought stress results from an unexpected deviation in climate, year to year depletion of groundwater/freshwater, and rapid population growth coupled with modernization and anthropogenic activities^3,4^. Crop productivity mainly depends upon the availability of freshwater, and drought stress reflects insufficient availability of water to plants. Consequently, it is a substantial barrier in achieving the goal to double agricultural crop production by 2050 to meet the expected demand of a rising global population^5,6^. At the cellular level, drought stress provokes the generation of reactive oxygen species (ROS), abscisic acid (ABA) and reactive nitrogen species (RNS) such as nitric oxide (NO), and its derived species including S-nitrosothiols (SNO) or S-nitrosoglutathione (GSNO) in plants^7–10^. NO is a small signaling molecule and it plays a crucial role in diverse plant cellular functions including plant defense, stomatal regulation, root development, etc., under stress and non-stress conditions^11^. NO can counteract the effects of ROS either by directly scavenging them or by re-stimulating the antioxidant defense system. However, cross-talk of NO and ROS modify NO’s benign property into a nitrosative agent, when its endogenous concentration along with duration of cross-talk is higher than appropriate causing nitrosative or nitro-oxidative stress in plants^12^. It is a well-known phenomenon that excess ROS induces oxidative stress in plants by disturbing their physiological and metabolic status including inhibition of mineral nutrient uptake, allocation and assimilation. Among various nutrients, nitrogen (N) is a key nutrient for plant growth and development as a building block of fundamental biological molecules, such as nucleotides, amino acids, proteins, ATP, co-enzymes and chlorophyll^13^. In fact, N content and its metabolism in and outside of plants plays an essential role as a signal in closely regulating the response associated with the resistance/tolerance/adaption of plants to environmental challenges, including drought stress^14,15^. Several studies including the present work have reported that drought stress affects N absorption and consequently inhibits enzymes through alteration in the transcriptional levels of transporters implicated in N-metabolism (e.g., nitrate (NO_3_^−^) transporters (NRTs), ammonium (NH_4_^+^) transporters (AMTs), nitrate reductase (NR), nitrite reductase (NiR), glutamine synthetase (GS), glutamate synthase (GOGAT), glutamate dehydrogenase (GDH))^16–18^.

**Figure 1:**
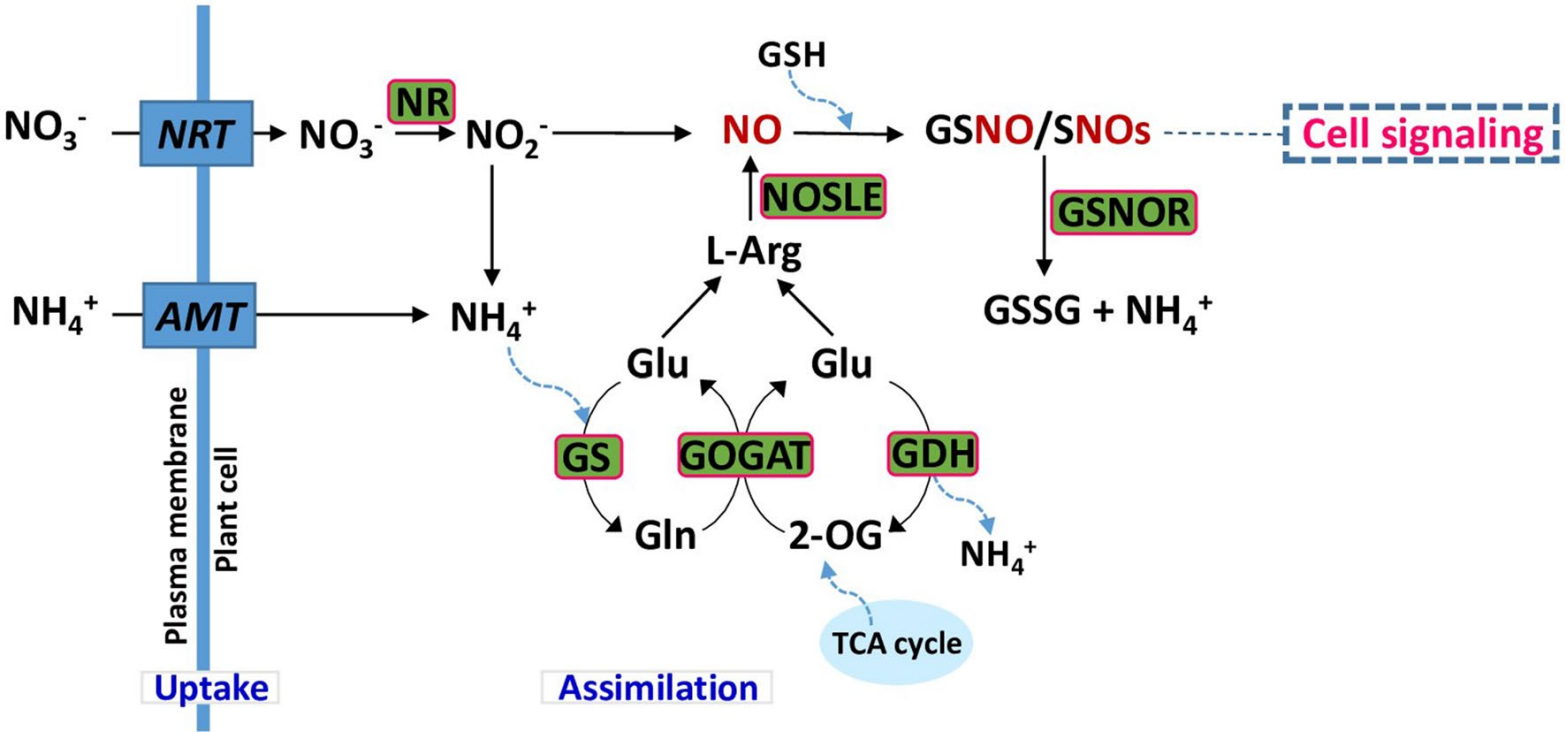
Nitrogen uptake and assimilation, and its interconnection with nitric oxide signaling process in plants.

The N-assimilation process begins with the uptake of inorganic NO_3_^−^, and ends with the final assimilation into organic compounds or amino acids with intermediate synthesis of NO_2_^−^ and NH_4_^+^ by the catalytic activities of NR and NiR, respectively. Before the conversion of NH_4_^+^ directly to amino acids, it can also be reduced to NO via the glutamine synthase-glutamine-2-oxoglutarate amino transferase (GS-GOGAT) system. After that, as a part of a defense mechanism NO may react with reduced glutathione (GSH) to produce SNO or GSNO, which is a major stable reservoir of NO in the plant cell^19^. GSNO is then metabolized by its conversion into oxidized glutathione (GSSG) and NH_4_^+^ by NAD(P)H dependent enzyme GSNO reductase (GSNOR)^20^ (Fig. 1). This enzyme is responsible for maintaining NO homeostasis and protecting the plant cell from nitrosative or nitro-oxidative stress. Studies suggest that N-assimilatory enzymes are the significant determinants in NO-mediated improved plant growth by fine-tuning of NO synthesis and scavenging^20,21^. Hence, N–NO regulation responses in plants have been considered crucial, as NO is synthesized during N assimilation via enzymatic and non-enzymatic pathways. The enzymatic reductive pathways include: nitrite-dependent reactions via xanthine oxidoreductase (XOR), NR, nitrite dependent nitric oxide reductase (Ni:NOR). The oxidative route includes l-Arginine (l-Arg)-dependent nitric oxide synthase like enzyme (NOSLE) activity, and polyamines or hydroxylamines reactions.

It has been highlighted that NO interacts with various phytohormones such as ABA, auxin, cytokinin, gibberellins, salisylic acid, brassinosteroid, ethylene, jasmonic acids, and polyamines^22^. Amongst all, ABA is the best-known stress phytohormone that regulates various key physiological responses to drought stress. Interestingly, NO is an intermediate signaling molecule of these responses. As such, NO helps to pass the ABA-induced signaling events in plants to respond accordingly during stress conditions. Studies revealed that NO and ABA are interlocking molecules, and signal one another to synthesize for subsequent change in redox balance and development of redox homeostasis in the plant cell. Hence, NO and ABA facilitates adaptive/tolerant responses under stress^23^. However, NO-dependent ABA-induced physiological responses are not always the result of cooperative/synergistic interaction between NO and ABA. In fact, NO may also be an antagonist and exert downstream regulation to ABA in order to finely tune the ABA-triggered responses^24,25^. Several factors including plant developmental stage, tissue- and time-dependent pattern of accumulation, threshold levels, interaction with other hormones, and the environmental interactions determine this diverse functional relationship between NO and ABA.

It is worth mentioning that most studies have shown that individually applied exogenous NO or ABA alleviates various abiotic stresses, including drought stress. To date, however, only a few studies regarding their co-mediating effects are available, and most of these focus only on physiological processes of seed dormancy, stomatal behavior, seed germination and root development^23,26^. Hence, not much is known about the possible interactive effects of both signaling molecules at the whole plant physiological level. Therefore, the main objective of the present study is to evaluate the effects of individual and simultaneous addition of NO and ABA under PEG-mediated drought stress using Indian mustard (*Brassica juncea*) as a model plant. *B. juncea* is mainly cultivated for its oil and leaves which constitutes various vital nutritional and medicinal applications. We chose this crop as a model because of its high photosynthetic capacity^27^, and the natural genetic variation in the traits related to drought tolerance. Various parameters of N uptake and metabolism (such as NO_3_^−^, NO_2_^−^, NH_4_^+^, NH_4_^+^/NO_3_^−^ ratio, total N and free amino acids content) were analyzed. Proline metabolism, mineral nutrients status, and enzymatic and non-enzymatic NO synthesis along with expression profile of N-uptake and assimilating genes were also assessed in this study. As plants face multiple stresses at a time, individual study of similar genes under different stress conditions will further help to discover the role of some specific genes which might be regulated by abiotic stress leading to the development of improved mustard varieties. The gene expression study of N-related transporters will be of particular importance for generating new *Brassica* varieties with increased N use efficiency and tolerance to drought stress through transgenic approaches. In the present work, we elucidated underlying mechanism to NO and/or ABA crosstalks in drought stress with particular reference to N-metabolism, and related changes at molecular and biochemical levels that drive modulation in oxidative stress.

## Results

Results are discussed by comparing the control plants with PEG or a group of nitrate agonists (i.e., PEG + ABA, PEG + NO and PEG + NO + ABA) rather than with NO or ABA alone, because ABA or NO treatments were not significantly different to control (no water stress) for all parameters except SNO/GSNO content and some micro-macronutrients studied (Supplementary Materials Table S1). Further, we have grouped all three PEG-combination treatments together as “nitrate agonists” as they showed similar trends of responses for all parameters studied; however, the magnitude of their effect was different. The effect of PEG + ABA was significantly less than PEG + NO for all parameters except for endogenous NO and its stable reservoir, i.e., SNO and GSNO content. PEG + ABA treatment was found to be equally effective to PEG + NO only when it was supplied together with NO (i.e., PEG + NO + ABA).

### Effect of water stress on growth, biochemical and physiological parameters, and transcript levels

**Figure 2:**
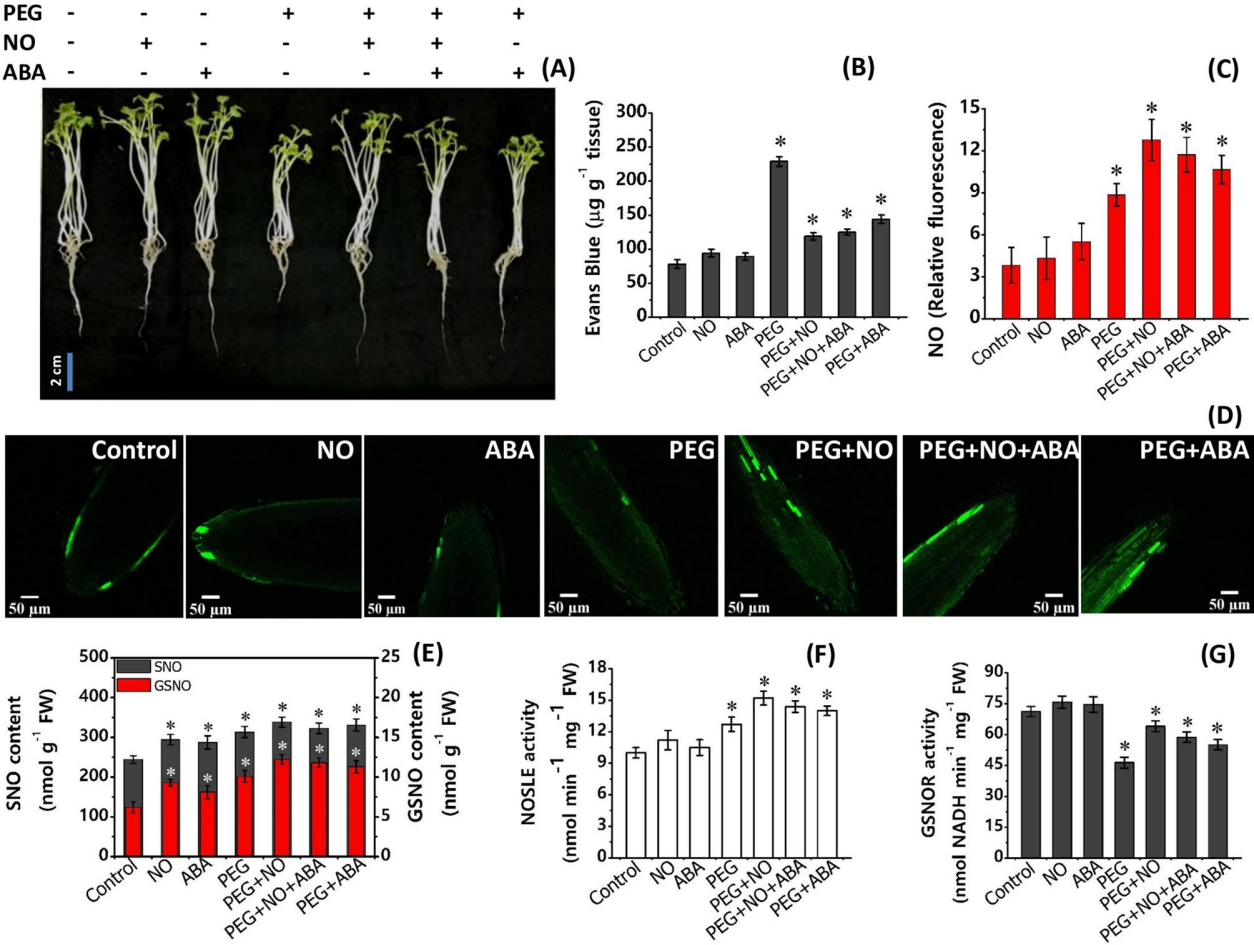
Phenotypic changes, oxidative stress, and NO-metabolism of 7-d-old *B. juncea* L. after treatment with PEG (10%) and its combination with NO (100 µM) and ABA (10 µM) (nitrate agonists) for 96 h. (**A**) Shoot and root phenotypic changes (Scale bar = 2 cm), (**B**) Evan’s blue uptake, (**C**, **D**) endogenous accumulation of NO (Scale bar = 50 µm) (**E**) S-nitrosothiol (SNO) and S-nitrosoglutathione (GSNO) content, (**F, G**) activity of L-Agr-dependent nitric oxide synthase-like enzyme (NOSLE) and S-nitrosoglutathione reductase (GSNOR). Values are mean ± S.E. of three independent experiments, each including four biological replicates. Asterisk (*) indicates significant difference compared with control (*p < 0.05).

As depicted in Fig. 2A and Table 1, the phenotypic appearance (particularly root length) and growth parameters (such as shoot and root length, fresh and dry biomass) of 7-day-old Indian mustard seedlings were influenced negatively by 96 h exposure to PEG treatment. A significant reduction was noted in root length compared to shoot length under PEG treatment over control (Table 1). Furthermore, water stress caused membrane damage as the Evans Blue uptake was considerably higher in PEG treated roots, compared to control (Fig. 2B, Supplementary Materials Table S2). The analysis of endogenous NO and its stable reservoir, i.e., SNO and GSNO, is displayed in Fig. 2C–E. The PEG exposed roots showed a significant increase in CLSM-measured relative fluorescent intensity compared to control. A similar result was observed for total SNO and GSNO content. The activities of enzymes involved in NO synthesis and its degradation were also measured to assess NO homeostasis (Fig. 2F, G). After 96 h exposure of PEG-induced drought stress to 7-d old *Brassica* roots, the activity of L-Arg dependent NOS-like enzyme (NOSLE) was found to be increased by 27% over control. In contrast, GSNOR activity (responsible for NO homeostasis) was significantly decreased by ~ 30% upon PEG treatment over control (Supplementary materials Table S2).

**Table 1:** Effect of PEG and its combination with NO and ABA (nitrate agonists) on length (shoot and root) and plant fresh and dry weight of 7-days-old *B. juncea* L. seedlings after 96 h exposure to the treatments.

Treatments	Shoot length (cm)	Root length (cm)	Fresh weight (g)	Dry weight (g)
Control	8 ± 0.46^bc^	5 ± 0.25^b^	0.077 ± 0.003^b^	0.0057 ± 0.0001^ab^
NO	9.1 ± 0.53^a^	6.2 ± 0.30^a^	0.105 ± 0.004^a^	0.0071 ± 0.0002^a^
ABA	8.9 ± 0.52^ab^	6 ± 0.31^ab^	0.095 ± 0.003^ab^	0.0062 ± 0.0002^ab^
PEG	6 ± 0.32^e^	3 ± 0.14^c^	0.036 ± 0.001^e^	0.0024 ± 0.0001^c^
PEG + NO	8.2 ± 0.47^bc^	6 ± 0.25^a^	0.065 ± 0.002^c^	0.0060 ± 0.0002^ab^
PEG + NO + ABA	7.8 ± 0.44^cd^	5.8 ± 0.25^a^	0.055 ± 0.002^d^	0.0049 ± 0.0001^b^
PEG + ABA	7 ± 0.39^d^	5 ± 0.20^b^	0.055 ± 0.002^d^	0.0048 ± 0.0001^b^

Values are mean ± S.E. of three independent experiments, each including four biological replicates. Different letters within the same column represent significant difference between treatments, where “a” corresponds to the highest value and “d” or “e” to the lowest value.

**Figure 3:**
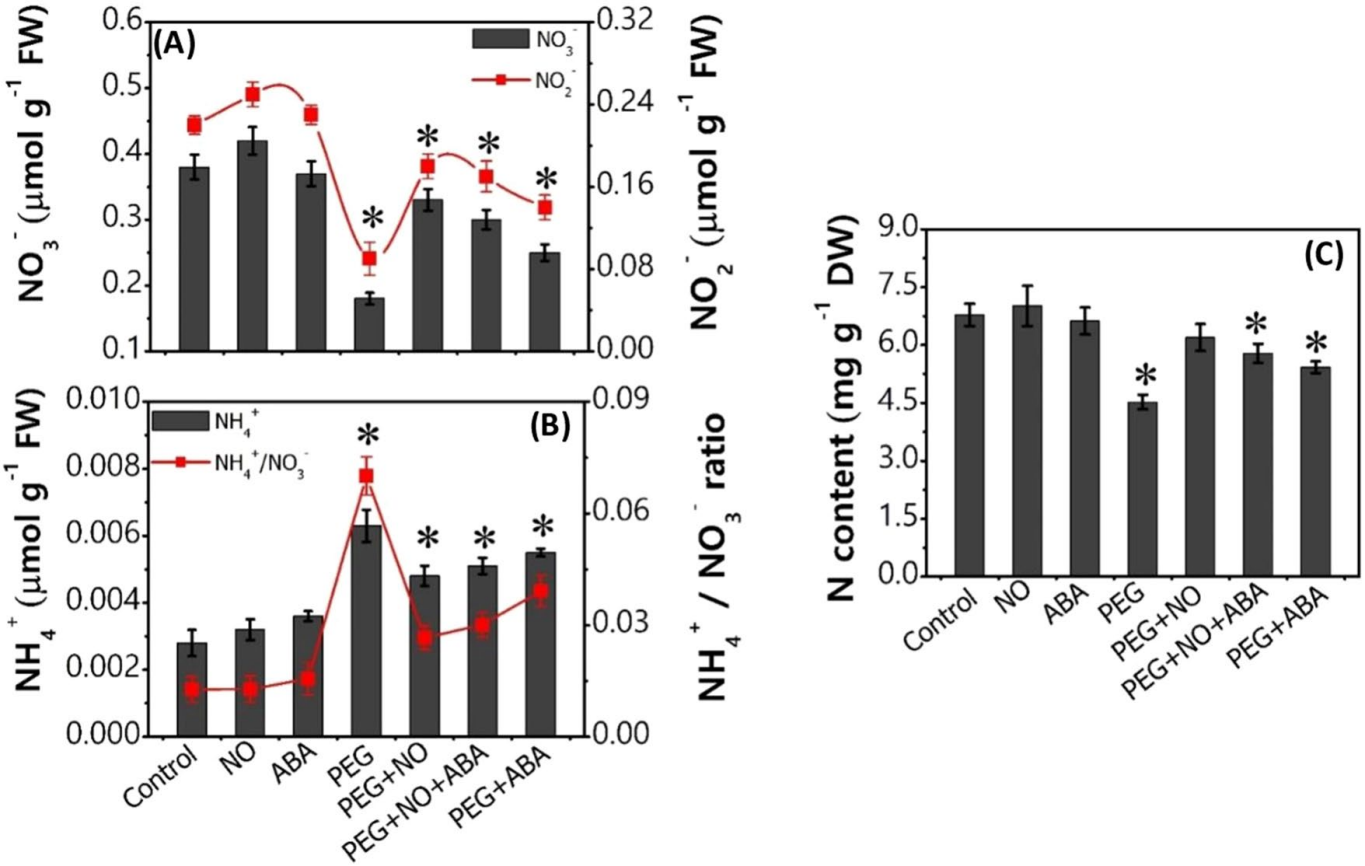
Content of nitrate (NO_3_^−^), nitrite (NO_2_^−^), ammonium (NH_4_^+^), ammonium- nitrate ratio (NH_4_^+^/ NO_3_^−^), and total nitrogen (N) of 7-day-old *B. juncea* L. roots after treatment with PEG (10%) and its combination with NO (100 µM) and ABA (10 µM) (nitrate agonists) for 96 h. Values are mean ± S.E. of three independent experiments, each involving four biological replicates. Asterisk (*) indicates significant difference compared with control (*p < 0.05).

**Figure 4:**
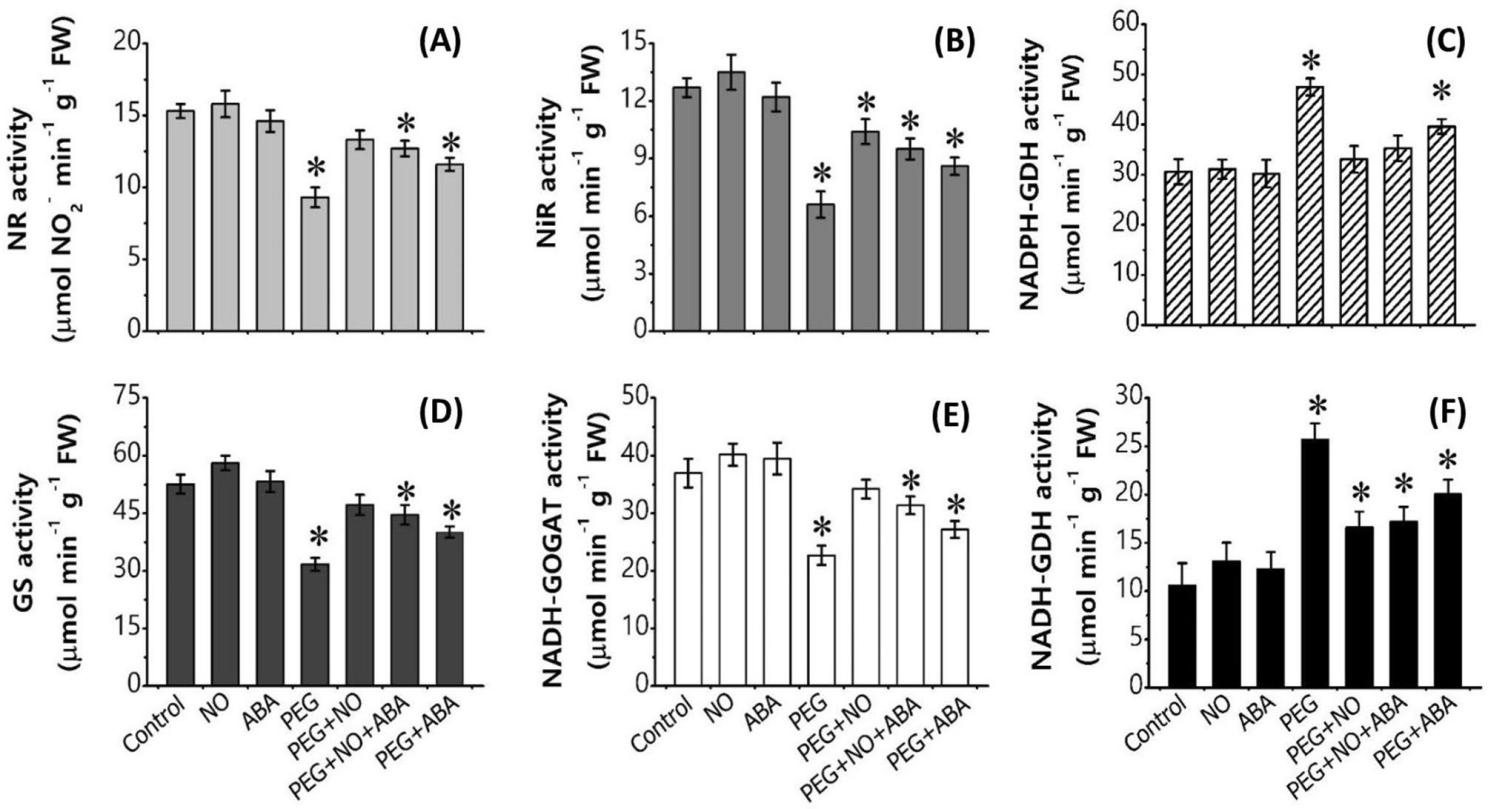
Activity of nitrate reductase (NR), nitrite reductase (NiR), glutamine synthetase (GS), glutamate synthase (GOGAT) and glutamate dehydrogenase (NADPH-GDH and NADH-GDH) of 7-day-old *B. juncea* L. roots after treatment with PEG (10%) and its combination with NO (100 µM) and ABA (10 µM) (nitrate agonists) for 96 h. Values are mean ± S.E. of three independent experiments, each including four biological replicates. Asterisk (*) indicates significant difference compared with control (*p < 0.05).

**Figure 5:**
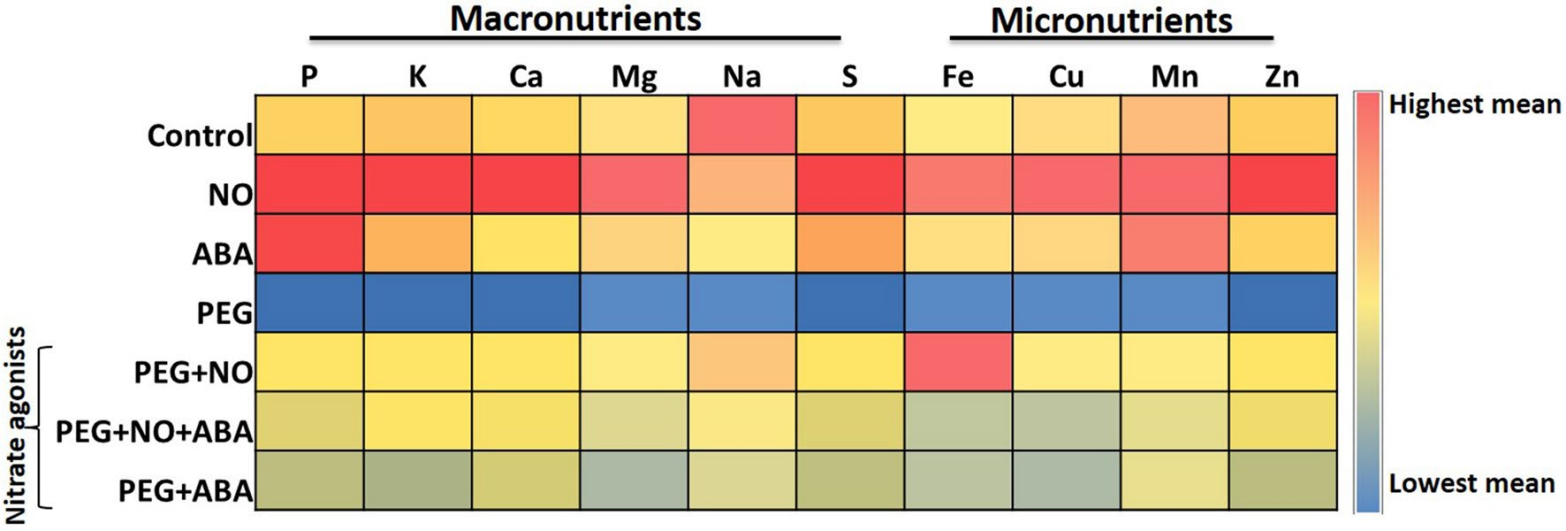
Heat map represents ICP-MS content of macro (P, K, Ca, Mg, Na, and S) and micronutrients (Fe, Cu, Mn, and Zn) of 7-d-old *B. juncea* L. roots after treatment with PEG (10%) and its combination with NO (100 µM) and ABA (10 µM) (nitrate agonists) for 96 h. Values are mean ± S.E. of three independent experiments, each including four biological replicates.

PEG-simulated drought stress disturbed the normal N absorption and its assimilation, causing a decrease in NO_3_^−^, NO_2_^−^, and total N content by ~ 52%, ~ 59%, and ~ 33%, respectively over control (Fig. 3A–C, Supplementary materials Table S2). In contrast, NH_4_^+^ content was significantly increased (1.25 times) which led to an increased NH_4_^+^/NO_3_^−^ ratio (4.5 times) in PEG treated *B. juncea* roots in respect to control roots (*p* < 0.05). The results related with the changes in activity of NR, NiR, GS, GOGAT and GDH enzymes have been portrayed in Fig. 4A–F. Under PEG solution, the root activity of NR decreased by ~ 39%, NiR by ~ 48%, GS by ~ 39%, and GOGAT by ~ 38%, over control values. In contrast, the activity of NADPH-GDH and NADH-GDH showed a significant increase under PEG treatment (*p < 0.05, Fig. 4C,F, Supplementary Materials Table S3). Also, as depicted in Fig. 5, PEG-drought stress exhibited a significant reduction in the accumulation of various macro- and micro-nutrients including phosphorus (P), potassium (K), calcium (Ca), magnesium (Mg), sodium (Na), sulphur (S), iron (Fe), copper (Cu), manganese (Mn), and zinc (Zn) (Supplementary materials Table S4). Furthermore, the analysis of total free amino acids, proline content and proline synthesizing enzyme (P5CS) activity showed a significant difference between control and PEG at *p* < 0.05. PEG treatment caused an increase in total free amino acids, proline and P5CS activity along with a decrease in the activity of ProDH over control treatment (Table 2). The percent (%)-increase or decrease values in all biochemical and physiological parameters under control versus all treatments are given in Supplementary Materials Table S2.

**Table 2:** Effect of PEG and its combination with NO and ABA (nitrate agonists) on total free amino acid, proline content, and activity of ∆^1^-pyrroline-5-carboxylate synthetase (P5CS) and proline dehydrogenase (ProDH) of 7-days-old *B. juncea* L. roots after 96 h exposure to the treatments.

Treatments	Total free amino acids (mg g^−1^ FW)	Proline content (µmol g^--1^ FW)	P5CS activity (nmol NADPH oxidised min^−1^ mg^−1^ protein)	ProDH activity (nmol NADH formed min^-1^ mg^−1^ protein)
Control	6.9 ± 0.301^d^	0.329 ± 0.045^e^	30.4 ± 1.37^d^	170.0 ± 8.92^a^
NO	7.4 ± 0.333^cd^	0.317 ± 0.033^e^	29.5 ± 1.84^d^	173.6 ± 7.33^a^
ABA	7.0 ± 0.300^d^	0.321 ± 0.042^e^	29.1 ± 1.67^d^	170.7 ± 7.77^a^
PEG	12.7 ± 0.533^a^	0.452 ± 0.040^a^	48.8 ± 1.61^a^	121.3 ± 6.45^e^
PEG + NO	8.2 ± 0.411^c^	0.401 ± 0.029^c^	35.2 ± 1.46^c^	158.3 ± 7.25^b^
PEG + NO + ABA	8.8 ± 0.412^c^	0.361 ± 0.035^d^	39.6 ± 1.33^b^	150.2 ± 7.17^c^
PEG + ABA	9.6 ± 0.431^b^	0.433 ± 0.042^b^	41.1 ± 1.35^b^	139.4 ± 5.83^d^

Values are mean ± S.E. of three independent experiments, each including four biological replicates. Different letters represent significant difference between treatments, where “a” corresponds to the highest value and “d” or “e” to the lowest value.

**Figure 6:**
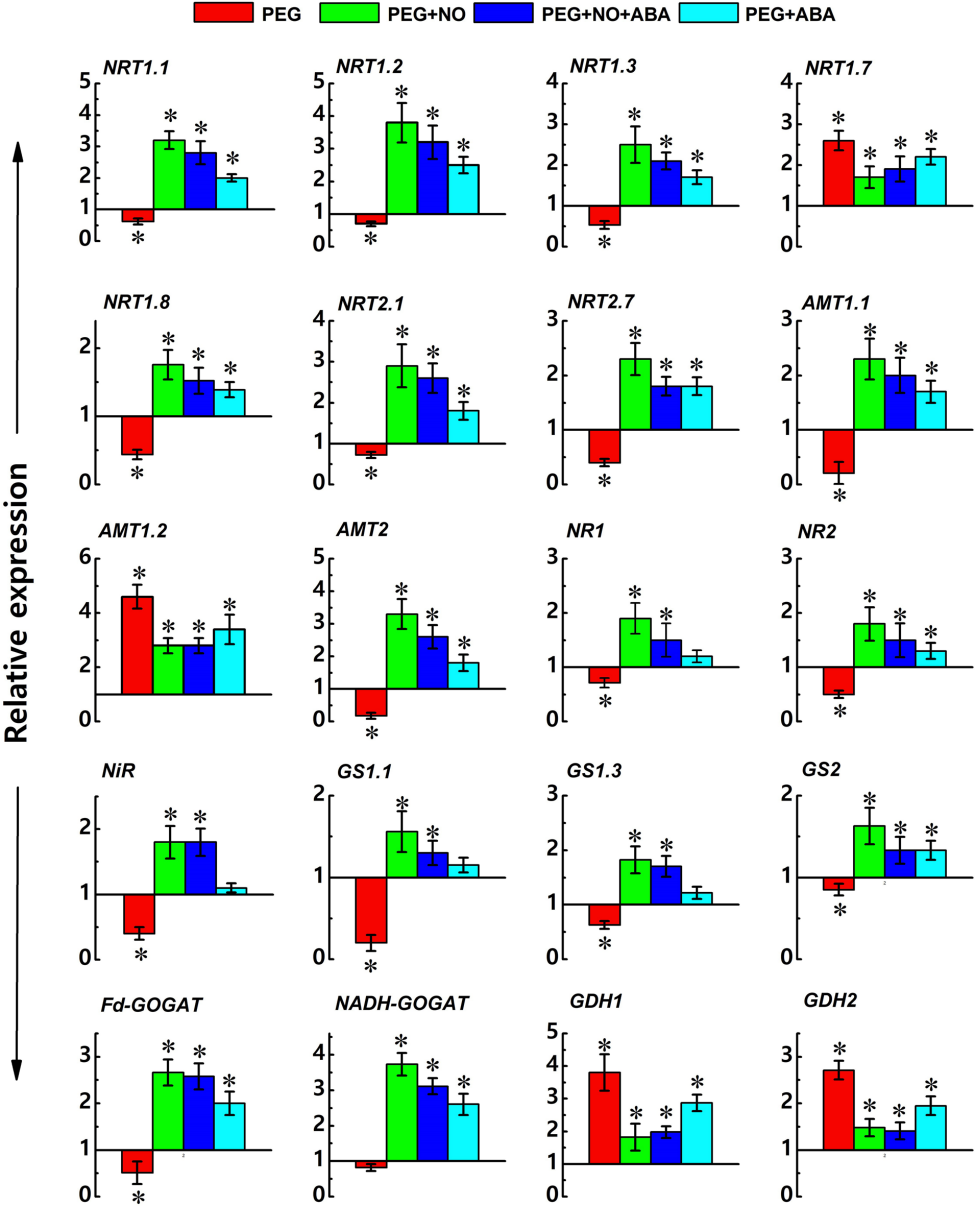
Relative expression levels of key genes related to nitrogen absorption and assimilation including nitrate transporter (*BjNRT*), ammonium transporter (*BjAMT*), nitrate reductase (*BjNR*), nitrite reductase (*BjNiR*), glutamine synthetase (*BjGS*), and glutamate synthase (*BjGOGAT*), and glutamate dehydrogenase (*BjGDH*) in 7-days-old *B. juncea* L. roots after treatment with PEG (10%) and its combination with NO (100 µM) and ABA (10 µM) (nitrate agonists) for 96 h. Expression values were calculated relative to the expression of plants grown in control condition under normalization with *actin* gene (2^−ΔΔCT^). Values are mean with CIs of three independent experiments, each including four biological replicates. *p < 0.05 compared with control indicates significant difference.

The expression profiles of genes associated with N-uptake are shown in Fig. 6 and Table S8, where the results are shown as relative fold changes in PEG and nitrate agonist treatments with respect to control values. Treatments including NO and ABA alone were not significantly different and hence are not discussed while explaining the results with respect to control. The fold change values are given in Supplementary Materials Table S5. After 96 h of PEG treatment, the expression of genes encoding for NRT (*BjNRT1.1, BjNRT1.2, BjNRT1.3, and BjNRT1.8), NRT2 (BjNRT2.1 and BjNRT2.7*), and AMT (*BjAMT1.1*) were down-regulated except for *BjNRT1.7* and *BjAMT1.2* which were up-regulated, respective to control. Similarly, all N-assimilation related genes encoding NR (*BjNR1* and *BjNR2*), NiR, GS (*BjGS1.1, BjGS1.3* and *BjGS2*), and GOGAT (*BjFd-GOGAT* and *BjNADH-GOGAT*), except GDH (*BjGDH1* and *BjGDH2*) under PEG alone treatment were also down-regulated (Fig. 6). Further, out of a total of 20 genes expressed, four genes were up-regulated and 16 genes were down-regulated in PEG alone treatment (Fig. 6).

### Effect of application of nitrate agonists on growth, biochemical, physiological and transcript levels under PEG-water stress

Measurements of the growth parameters showed that plants were able to withstand PEG-induced water stress better when supplied with nitrate agonist treatments. As such, PEG + NO, PEG + NO + ABA, and PEG + ABA treatments resulted in a significant recovery in PEG induced loss of shoot length, root length, fresh biomass, and dry biomass compared to PEG alone (Fig. 1A, Table 1). Among three nitrate agonists, PEG + ABA is comparatively less effective than PEG + NO, while application of PEG + ABA co-supplied with NO i.e., PEG + ABA + NO is equally effective to PEG + NO. Further, these nitrate agonist treatments caused a significant modulation of observed changes in membrane damage by reducing the PEG evoked increase in Evans Blue uptake (Fig. 2B). The endogenous NO content (relative fluorescent intensity) of *B. juncea* roots was comparatively higher in all three nitrate agonist treatments compared to PEG alone. A non-significant increase was noted for total SNO, whereas GSNO levels were significantly higher in NO containing nitrate agonist treatments (PEG + NO and PEG + NO + ABA) over PEG alone (Fig. 2C-E). Moreover, plants supplied with nitrate agonists also resulted in an increase of NOSLE activity, in fact more than PEG alone, with a greater increase under PEG + NO treatment (Supplementary Materials Table S2). Activity of GSNOR increased under PEG-combination treatments compared to PEG alone, although the activity was lower in respect to control. Compared to PEG alone, nitrate agonist treatments supplementation recovered the PEG-induced loss in the contents of nitrogenous compounds (NO_3_^−^, NO_2_^−^ and total N), (Fig. 3A–C), the activity levels of N-uptake and assimilation enzymes (Fig. 4A–D), and macro- and micro-nutrients (Fig. 5, Supplementary Materials Table S4). Among nitrate agonist treatments, PEG + NO showed maximum recovery when compared to PEG + NO + ABA and PEG + ABA. Also, it was noticed that the application of PEG + NO treatment significantly modulated or prevented the PEG-induced changes in NADPH-GDH and NADH-GDH activities (Fig. 4E, F), total free amino acids, proline level and P5CS activity (Table 2) towards the control level. It was noticed that ProDH activity was remarkably increased under PEG + NO, PEG + NO + ABA, and PEG + ABA treatments compared to PEG treatment alone. The percent (%)-increases or decreases in all biochemical and physiological parameters under PEG versus nitrate agonist treatments are given in Supplementary Materials Table S2.

Nitrate agonist treatments countered the PEG-triggered up- and down-regulation of gene expressions. PEG-induced down-regulated expression of nitrate transporters was found to be up-regulated even more than the control under the action of nitrate agonist treatments. The expression was increased many times more in PEG + NO than PEG + ABA treatment compared to control and PEG alone for *BjNRT1.1*, *BjNRT1.2*, *BjNRT1.3*, *BjNRT1.8*, *BjNRT2.1*, *BjNRT2.7*, *BjAMT1.1*, and *BjAMT2* (Fig. 6, Supplementary Materials Table S5). Similarly, the expression was also higher for N-assimilation transporters such as *BjNR1* and *BjNR2, NiR*, *BjGS1.1, BjGS1.3, BjGS2, BjFd-GOGAT* and *BjNADH-GOGAT* in *B*. *juncea* roots supplied with nitrate agonist treatments. PEG-induced expression levels of *BjNRT1.7*, *BjAMT1.2, BjGDH1* and *BjGDH2* were lower than the treatments of nitrate agonist which were relatively lower than PEG alone but still higher than control. Taken together, the transcripts of all genes were higher in PEG + NO compared to control/PEG/PEG + ABA treatments except PEG + NO + ABA treatment. Further, out of a total of 20 genes expressed, the expression of 4 genes were commonly up-regulated and no gene was found to be commonly down-regulated in PEG and nitrate agonist treatments together (Fig. 6).

## Discussion

The real-time CLSM imaging of the endogenous NO accumulation showed that green relative fluorescence intensity was comparatively higher in PEG and nitrate agonists treated roots than control roots. However, the intensity of endogenous NO was not significantly higher in SNP (NO donor) treated roots than control roots. The reason may be ascribed to that (1) after accumulation NO might have converted into its most stable reservoir species i.e., SNO/GSNO as it is evident from Fig. 2E where the content of SNO/GSNO under NO alone is significantly higher than control. (2) higher NO synthesis occurs when a plant perceives the stress condition either due to oxidative or nitro-oxidative stress which was not exhibited by 100 µM SNP-treated roots in the present study. The result was validated/supported by our previous finding that an increase of spectrophotometrically determined NO content and ABA content under PEG treatment, with more increase under PEG-combined with NO and/or ABA treatments in *B. juncea*^28,29^. Our result was also supported by the study of Cao et al.^30^, who observed that NO production level was higher in SNP + PEG treated roots of rice seedlings. The higher endogenous NO level in nitrate agonist treatments compared to PEG appeared to be beneficial to the plant as an improvement in growth parameters and phenotypic changes particularly root morphology was observed against PEG-water stress.

The maximum increase in growth biomass was noted in seedlings supplied with PEG + NO compared to PEG + ABA treatment. This is probably linked to more accumulation of NO and its signaling cascade role leading to the development of root hairs, thereby increased root surface area, improved water uptake i.e., leaf relative water content, (data is shown in our previous study^28^) and other nutrients, as evident from increased levels of macro-micronutrients and plant growth in the present study. The role of exogenous NO and ABA application to enhance different growth parameters has also been demonstrated in many crops under drought stress^31–33^.

Furthermore, exogenous 10 µM ABA treated plants exhibited more growth particularly shoot growth compared to control plants. ABA is generally regarded as an inhibitor of shoot growth, however, this view contradicts our finding and also many other published reports of stimulatory effects of ABA under non-stress conditions^34–37^. Studies have shown that wild types with higher endogenous ABA level or ABA-deficient mutants are comparatively dwarfed with smaller leaf size in several plant species^38,39^, suggested that the inhibitory effect of ABA depends upon the ABA applications, tissue altered physiological ABA level and corresponding developmental changes, and tissue sensitivity in crop plants^40–43^, however, the scientific discussion is still lagging^43^. The effect of ABA application at 50 µM and 100 µM in inhibiting shoot growth in *B. juncea* cv. Varuna in well-watered condition has previously been reported in our study^44^.

Proline and total free amino acids are crucial to N metabolism. Under the supplementation of nitrate agonist treatments, the level of total free amino acids was reduced but the concentration remained relatively higher than control plants, which is actually a part of tolerance/adaptive process contributing to osmotic balance under stress conditions like cold and water-deficit stress^45,46^. However, the combined effect of both on free amino acids response under various abiotic stresses has not been evaluated, until the current work. In the present study, PEG mediated up-regulation of P5CS activity with simultaneous down-regulation of ProDH accompanied higher accumulation of proline which is in agreement with other studies^17,47–49^. Reduction in proline level under nitrate agonist treatments was consistent with lower P5CS activity along with significant increase in ProDH activity. The result suggests that application of NO helps to maintain the proline level by manipulating its synthesis (P5CS activity) and catabolism (ProDH activity). Reduced proline level, as a consequence of NO donor treatment, has also been reported^50–52^. Several researchers agreed that a balance between proline synthesis and catabolism instead of its excessive accumulation plays a vital role in plant tolerance against drought stress^53,54^.

Macro and micronutrients such as N, P, K, S, Ca, Na, Mg, Fe, Zn, and Mn participate in metabolic processes and support the plant physiology and growth development^55^. Drought stress is known to interfere with the availability, uptake and distribution of essential nutrients in plants^56^, and causes a disturbance in physiological functions of plants. In the present study, nitrate agonist treatments surpassed the nutrient deficiency caused by PEG-water stress by increasing all the micro- and macro-nutrients. Among nitrate agonists, the effect of NO alone or together with ABA (PEG + NO or PEG + NO + ABA) was more efficacious than ABA alone under PEG (PEG + ABA) treatment, suggesting that NO participates in water stress acclimation responses to changes in nutrient availability, allowing better performance of the plant by alleviating oxidative stress. This might be related to higher endogenously generated NO level under PEG + NO and PEG + NO + ABA than PEG + ABA treatments. The endogenously generated NO content may have contributed to improve the plant’s capacity to absorb more nutrients from the growth medium mediated by its scavenging interaction with ROS or by stimulating the antioxidant system, as evidenced from lower O_2_^·−^, H_2_O_2_, and MDA, and higher enzymatic and non-enzymatic antioxidants activities in *B. juncea* cv. Varuna in our previous study^28^. Furthermore, it has been reported that deficiency of nutrients causes endogenous NO synthesis and degradation rates to trigger responses associated to stress acclimation^57,58^. The role of exogenous NO supplementation to enhance the micro-macronutrients status of crop plants has been studied^59,60^. However, the interactive action of NO plus ABA is not available in the literature.

S-nitrosation is the process of SNO or GSNO (mobile NO bioactivity reservoir) formation which have great physiological relevance in plant development and stress responses^20,61^. In the present study, decreased GSNOR activity under PEG and nitrate agonist treatments was associated with the increased content of GSNO/total SNO level, compared to control. The result is supported by previous experiments that showed a negative correlation between SNO/GSNO levels and GSNOR activity^62,63^. Further, decreased activity of GSNOR over control, but increased activity with respect to PEG alone under nitrate agonist treatments indicated that SNO maintains NO homeostasis by regulating the intracellular level of GSNO, and indirectly total SNO through their degradation into oxidized glutathione (GSSG) and NH_4_^+^ as a part of glutathione-based detoxification mechanism. Under nitrate agonist treatments, increase of GSNO/total SNO content with respect to PEG was directly related to the endogenous level of NO and promotion of GSH-dependent antioxidants capacity in the protection against oxidative stress^19,64^, as evident from better changes in growth phenotype of *B. juncea* seedlings in the present study. However, studies have shown that the SNO/GSNO accumulation does not always confer protection in fact it may be responsible for nitrosative stress^65,66^, which was not the case in our experiment. The reason may ascribed that SNO/GSNO exhibit different effects depending upon many factors such as its intracellular concentration, diverse abiotic stress, dissimilar treatment time period, and strength of stress applied to induce oxidative stress^67^.

NO is an N-based molecule and an end product of N-assimilation; therefore, we have determined whether or not NO and ABA affects N-metabolism, and also analyzed the interconnection between the intermediates of N-assimilation and NO signaling. The PEG-mediated water stress significantly impeded the root NO_3_^−^ content, which was positively correlated with down-regulated expression of *BjNRT1.1, BjNRT1.2, BjNRT1.3, BjNRT1.8, BjNRT2.1,* and *BjNRT2.7* transporters except *BjNRT1.7*. In *Arabidpsis*, *NRT1.7* (*AtNRT1.7*) functions as a source to sink remobilization of NO_3_^−^ in phloem^68^. PEG-induced up-regulation of *BjNRT1.7* might reflect their importance in plant adaptation to stress through its involvement in nitrate remobilization to nitrogen-demanding tissues/organs, which is important to sustain plant growth due to stress-induced loss of nitrogen uptake. It may also be a reason that *NRT1.7* might be down-regulated at early response of stress perception similar to *BjNRT1.5* which was up-regulated after 1 h and down-regulated after 24 h of abiotic stresses exposure which suggested to be an adaptive response to stress condition^69^. However, the kinetic expressions of *BjNRT1.7* are needed to be addressed in a future study for both roots and shoots which will contribute much to our understanding. Further, more experimental studies also need to be done in *B. juncea* on N-transporters under different stresses which are lacking in the literature. Water stress induced decreased NO_3_^−^ content indicating decreased NO_3_^−^ absorption by root cells, which could be due to overproduction of ROS that causes loss in plasma membrane integrity or electrolyte leakage^28,29^. However, a greater NO_3_^−^ content was observed when plants were grown with nitrate agonists, although, PEG + NO and PEG + ABA + NO were better than PEG + ABA. These results imply that a higher NO_3_^−^ uptake rate is related to NO regulated up-regulation of most NRTs genes, leading to water stress tolerance in the plant.

NR is the cytosolic enzyme of NO_3_^−^ assimilation and its activity was found to be sensitive (decreased) at 96 h of PEG treatment, which is similar to published results in some cases^70,71^, but not in others^30^. The reason may be related to the severity and duration of the stress imposed to the plants, where NR activity varied between tolerant and sensitive genotypes depending upon type of stress and treatment time^30^. Similar to NR, NiR activity was also decreased which was associated with reduced availability of NO_2_^−^ due to impaired NR-catalyzed NO_3_^−^ reduction under PEG-water stress. However, the nitrate agonist treatments restored the NR and NiR activities, indicating that NO and ABA facilitates the absorption and subsequent assimilation in roots. As a consequence, production of NO_3_^−^ and NO_2_^−^ were increased as evidenced by up-regulated expression of *BjNR1, BjNR2 and BjNiR* transporters. In spite of a decrease in NR and NiR activities by PEG-water stress, the NH_4_^+^ content was not affected, in fact, it increased significantly with respect to control. Similar results were obtained in other plants, with increased NH_4_^+^ content under drought stress, which may be associated with the plant’s tolerance mechanism^71^. Furthermore, the increase of NH_4_^+^ content was correlated with increased GDH activity (NADH-deamination and NADPH-amination) followed by its up-regulated expression (*BjGDH1* and *BjGDH2*), probably to compensate the inhibition in NiR activity and to provide the required NH_4_^+^ needed for amino acids synthesis. This is evident from an increased level of total free amino acids in response to PEG-water stress. The increase of NH_4_^+^ content was a result of up-regulated *BjAMT1.2* which plays an important role in enhancing NH_4_^+^ uptake and in acclimation to less water availability in response to water stress in our study. Subsequently, water stress suppressed GS and GOGAT enzymes which may cause excessive levels of NH_4_^+^ and in turn caused NH_4_^+^ toxicity in plant cells. The application of nitrate agonist treatments altered the NH_4_^+^ assimilation pathway, favored the enhancement of GS/GOGAT cycle and suppressed the amination and deamination of GDH pathway, which may contribute to maintaining the NH_4_^+^ conversion to glutamine and glutamate, and eliminate excess NH_4_^+^. As a result, in the presence of nitrate agonists, water stress had a lesser effect on NH_4_^+^ assimilation which in turn improved total N content leading to better growth of *B. juncea* seedlings. Up-regulated *BjGS1.1, BjGS1.3, BjGS2, BjFd/NADH-GOG* and down-regulated *BjGDH1* and *BjGDH2* under nitrate agonists application were consistent with increased activity of GS/GOGAT, and decreased activity of GDH compared to PEG alone, respectively. Goel and Singh^69^ have also reported that most of the genes of N-uptake and assimilation in *B. juncea* were down-regulated under salt, osmotic, cold and heat stress.

NR-mediated NO production is widely known with physiological and molecular mechanism, however, the existence of an l-arginine-dependent NOSLE activity has also been proposed as a possible source of NO generation in plant that is similar to NOS activity of animals, although the molecular mechanism responsible for this activity has not been identified so far in higher plants^72,73^. In the present study, the inverse relationship between NR activity and NO accumulation (lower NR activity and higher NO content) coincided with NOSLE activity, and possibly played a crucial role in synthesizing NO under PEG-triggered oxidative stress, as suggested by Hancock^74^. Although in nitrate agonist treatments a further increase in NOSLE activity had the trend similar to the increased NO content, indicating that increased NO_2_^−^ level may also be a determinant of NO production by allowing higher NR activity to reduce NO_2_^−^ into NO. This is in agreement with other studies, and suggests that NO_2_^−^ production is itself linked to NR-mediated nitrite-induced NO production^21,75^.

## Conclusions

**Figure 7:**
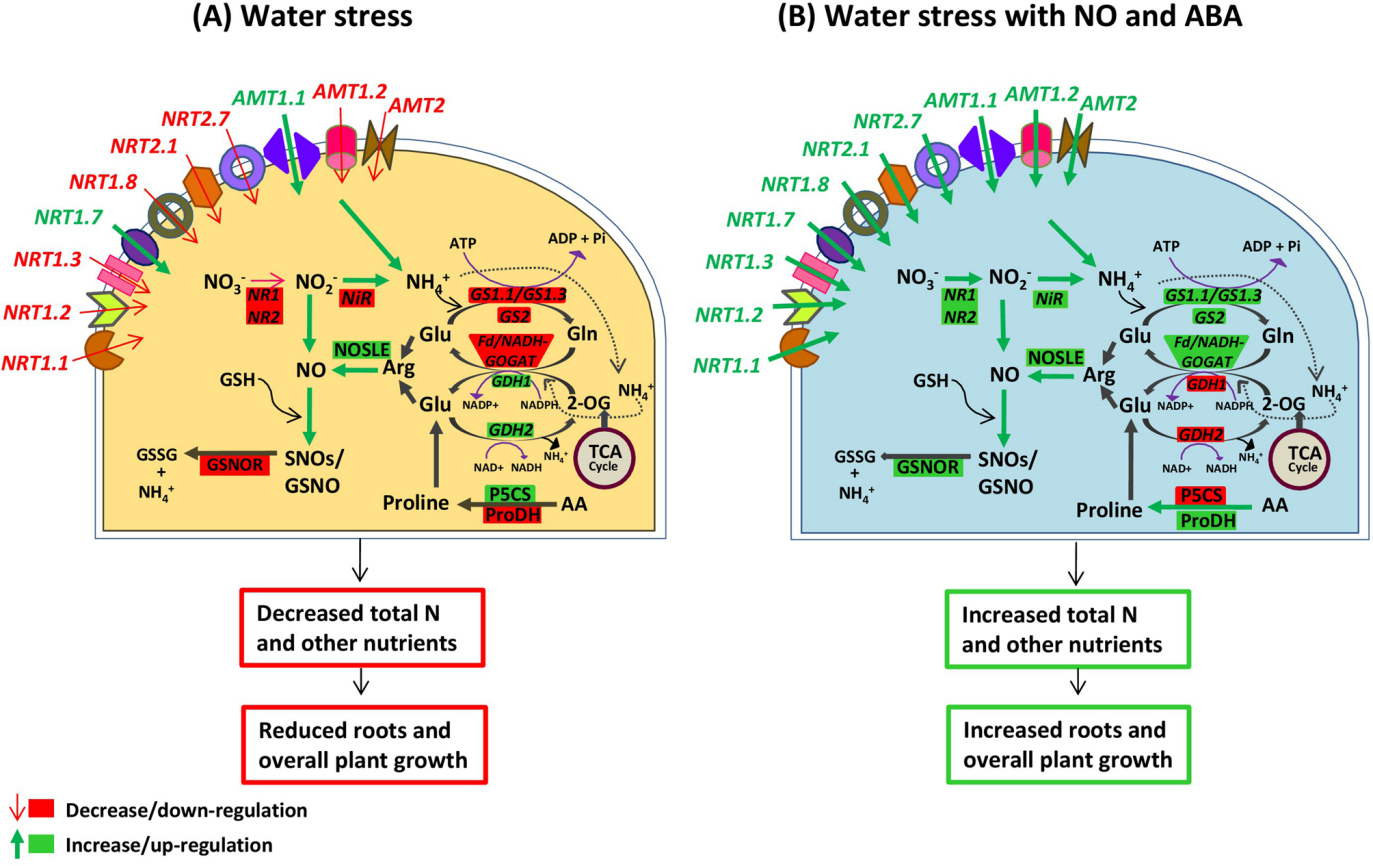
A schematic comparison of (**A**) PEG-water stress and (**B**) nitrate agonists regulated N–NO coordinated underlying physiological phenomenon that shows the relationship of NO generation and scavenging to molecular mechanism of N-assimilation in *B. juncea* L. roots. Red boxes and narrow arrows represent decrease or down-regulation, while green boxed and wide arrows indicate increase or up-regulation in N–NO pathway. *NO*_*3*_^*−*^ nitrate, *NO*_*2*_^*−*^ nitrite, *NH*_*4*_^*+*^ ammonium, *SNO* S-nitrosylated glutathione, *GSNO* S-nitrosoglutathione, *NO* nitric oxide, *Glu* glutathione, *AA* amino acids, *GSSG* oxidised glutathione, *P5CS* ∆^1^-pyrroline-5-carboxylate synthetase, *ProDH* proline dehydrogenase, *NR* nitrate reductase, *NiR* nitrite reductase, *NOSLE* l-arginine dependent NOS-like enzyme activity, *GSNOR* S-nitrosoglutathione reductase, *GS* glutamine synthetase, *GOGAT* glutamate synthase, *GDH* glutamate dehydrogenase.

The results obtained in the present study are summarized through a hypothetical model (Fig. 7). The figure shows a putative mechanism underlying cross-regulation between N metabolism and NO/+ABA signaling under drought stress through a gene expression approach, followed by targeted physiological analysis in *B. juncea* cv. Varuna. Overall, we have demonstrated that NO and ABA mediated drought tolerance is associated with greater total N content along with other mineral nutrient availability, increased N-metabolism enzymes activities, and differential regulated expression of *NRT*, *AMT*, *NR*, *NiR*, *GS*, *GDH* and *GOGAT* genes in *B. juncea.* It is clear from the data that NO either alone or together with ABA had an ameliorating effect on drought toxicity on growth attributes, manifested in term of increased biomass under PEG treatment. The improved growth could be a result of increased but balanced accumulation and interaction of NO level and ABA level as a result of PEG + NO, PEG + NO + ABA, and PEG + ABA. In conclusion, our data revealed that NO-ABA signaling and N-assimilation interplays in intricate ways to improve drought tolerance.

## Materials and methods

### Plant material, growth conditions and stress treatment

Indian mustard (*B. juncea* L. *cv.* Varuna) plants were used in this study. Disease free seeds were provided by the Indian Agricultural Research Institute (IARI), Pusa, New Delhi. Seeds were surface sterilized for 2–3 min in 30% ethanol (v/v), and washed thoroughly several times with sterile water. Overnight soaked sterilized seeds were kept in the dark for germination at 25 ± 2 °C. After 2 days, germinated seedlings were grown up to seven days (d) hydroponically with 5% strength of Hoagland Medium (HM) solution (pH 6.2–6.5)^76^ in a growth chamber at 25 ± 2 °C with a day/night photoperiod of 16/8 h and relative humidity of 70%. Ten seedlings were maintained per PVC cup (fitted in a tray containing HM solution). 7-day-later, seedlings were supplied with sodium nitroprusside (SNP, a NO donor 100 µM) and ABA (10 µM) with or without polyethylene glycol 6000 (PEG 6000, drought stress inducer, 10%, Sigma, cat. no. 528877) treatment for 96 h, using different combinations: (i) Control (no water stress), (ii) 100 μM NO, (iii) 10 μM ABA, (iv) 10% PEG, (v) 10% PEG + 100 μM NO, (vi) 10% PEG + 10 μM ABA + 100 μM NO, and (vii) 10% PEG + 10 μM ABA. The last three treatments were grouped as “nitrate agonists” as they showed similar trends of N-related and other parameters studied; however, the magnitude of their effect was different particularly between PEG + NO and PEG + ABA. Aeration was maintained continuously and nutrient medium was replaced after every 2 days in order to keep seedlings away from nutritional deficiency. To prevent osmotic shock, PEG 6000 at 10% was added in HM solution to cause a gradual decrease in its osmotic potential until − 1.48 MPa, which is believed to cause low osmotic/water/drought stress toxicity. 10% PEG was selected as a moderate concentration based on the degree of drought susceptibility index, as reported in our earlier study^28^. The concentrations of ABA and NO along with 10% PEG were selected from our initial screening experiment carried out at various concentrations (0, 0.1, 1, 10, 50 and 100 μM ABA; and 0, 10, 50, 100, 150, 200 μM NO). Results showed that 10 μM ABA and 100 μM NO with 10% PEG were close to an optimum combination on analyzing shoot–root growth and other related developmental parameters in two *B. juncea* genotypes^44^. SNP was used as a constant source of NO releasing it in the nutrient medium. Although the concentration of SNP applied was not so high, but the SNP treatment containing trays were made photo-protective with aluminium foil to minimize the possibility of simultaneously releasing cyanide in the solution. After 96 h of treatments exposure, the roots of *B. juncea* seedlings were harvested, wrapped with tinfoil, and immediately frozen in liquid N_2_, and subsequently stored at − 80 °C until further analysis. The experiment was designed and treatments were arranged in a complete randomized block (CRB) design with four biological replicates (n = 4).

### Phenotypic changes and growth parameters

Growth parameters were analyzed using shoot–root length, fresh weight, and dry weight of fresh seedlings. Dry weight was recorded after oven-drying the seedlings overnight at 70 °C. The phenotypic changes were assessed through the differences in shoot and root length under different treatments.

### Biochemical analysis

#### Measurement of cell viability (Evan’s Blue uptake)

Cell viability (also known as loss of plasma membrane integrity/ cell death) was measured spectrophotometrically after application of Evan’s Blue, following the method of Ederli et al.^77^. Fresh root tissues (100 mg) were incubated for 20 min in 2 ml of 0.25% (w/v) Evan’s Blue dye (Sigma, cat. no. E2129) solution prepared in 0.1 M CaCl_2_ (pH 5.5) solution. After that, roots were washed thoroughly for 15 min with water or 0.1 M CaCl_2_ (pH 5.5) to wash out unbound dye from roots completely. The root tissue with trapped Evan’s Blue was then homogenized with 1 ml of 1% (w/v) aqueous sodium dodecyl sulphate (SDS) cell lysis buffer to release trapped Evan’s Blue from the root tissue. The homogenate was centrifuged at 14,000×*g* for 15 min. The optical density of the supernatant was determined at 600 nm using SDS as blank. Concentration of Evan’s Blue was estimated by referencing a standard curve.

#### Analysis of endogenous NO, total S-nitrosothiol (SNO) and S-nitrosoglutathione (GSNO)

Endogenously produced NO was detected using NO-specific fluorescent dye diaminofluorescein diacetate (DAF-2DA, Sigma, cat. no. 251505), as described by Luo et al.^78^. Fresh roots having fine root tips were incubated with 10 µM DAF-2 DA dissolved in freshly prepared 20 mM HEPES–NaOH buffer (pH 7.4) for 15–20 min in dark at 37 °C, followed by washing with same buffer (pH 7.4) twice for 30 min. Images were visualized using confocal laser scanning microscope (CLSM) imaging system (495 nm excitation and 515 nm emission wavelength) and then processed to quantify NO production as an average signal green fluorescent intensity using ImageJ program. Data was expressed in arbitrary unit (AU). Four roots from each treatment were measured, and repeated thrice for each experiment. The method based upon hydrolysis of S-nitrosylation in the presence of HgCl_2_ was used to analyze the total SNO in root tissues, as described by Frungillo et al.^79^. The content of GSNO was detected and quantified adopting the liquid chromatography-electrospray mass spectrometry (LC-ES-MS) method of Airaki et al.^80^, with some modifications. The content of SNO and GSNO were expressed in nmol g^-1^ FW. The detailed methodologies are given in supporting file for material and methods.

#### Analysis of nitrogenous compounds and total N nitrogen

Fresh roots (0.025 g) were homogenised in 1.5 ml of 20 mM HEPES buffer (pH 8.0), centrifuged at 10,000×*g* for 10 min at 4 °C, and the supernatant was used to analyze the nitrogenous compounds using nitro-salicylic acid method of Cataldo et al.^81^ for NO_3_^−^, the method of Snell and Snell^82^ for NO_2_^−^ and Molins-Legue et al.^83^ for NH_4_^+^ contents. The contents of nitrogenous compounds were expressed in µmol g^−1^ FW. Lindner’s^84^ method of acid-peroxide digestion was used for estimation of total N in over dried root powders. The N content was calculated against a standard curve of (NH_4_)_2_SO_4_ and expressed as mg g^−1^ DW. The methodology is given in detail in the supporting file for materials and methods.

#### ICP-MS analysis for macro–micro elements

Macro (P, K, Ca, Mg, and Na) and micro nutrients (Mn, Fe, Cu, Zn) were estimated using inductively coupled plasma- mass spectrometer (ICP-MS, Agilent 7900), following the protocol described by Masson et al.^85^. Dried roots powder (0.1 g) were pre-digested with 4 ml of concentrated HNO_3_:HClO_4_ (60:40%, v/v) ratio at 120 °C until there was no emission of brown nitrogen oxide gas, further digested with HClO_4_ at 180 °C until the solution turns transparent. The digested mixture was diluted with sterile water to a final volume of 40 ml, and the content of macro and micro elements were analyzed. Total S estimation was done according to Chesnin and Yien^86^. Dried powder of roots (0.1 g) was digested with 5 ml mixture of HNO_3_ and HClO_4_ in 85:15 ratio (v/v). To 2 ml final volume of digested solution with distilled water, sodium acetate buffer (pH 4.8), 50% glycerol and 20% barium chloride were added. Turbidity was measured at 470 nm using violet filter on spectrophotometer.

#### Estimation of total free amino acids, proline and proline metabolism enzymes

The Yokoyama and Hiramatsu’s^87^ ninhydrin method, with some modifications was used to measure total free amino acids in root extracts at 570 nm using 80% ethanol as blank against l-leucine (Sigma, cat. no. L8000) standard, as described in the supporting file for material and methods. Bates et al.^88^ method was used to measure proline content. The detailed methodology has been described in our previous report^28^. For estimating proline metabolism enzymes activities, root enzyme extract prepared in 100 mM Tris–HCl buffer (pH 7.4), 100 mM β-mercaptoethanol, 10 mM MgCl_2_ and 1 mM PMSF was used for the determination of ∆^1^-pyrroline-5-carboxylate synthetase (P5CS; EC 2.7.2.11) and proline dehydrogenase (ProDH; EC 1.5.99.8) activity, as the method described by Garcia-Rios et al.^89^ and Reno and Splittstoesser^90^, respectively. The activity was expressed as nmol NADPH oxidised/ NADH formed min^-1^ mg^−1^ protein, respectively. The detailed methodology is provided in the supporting file for materials and methods.

#### Activities of key enzymes of N-metabolism

For extraction and assay for nitrate reductase (NR) activity in root tissues (0.2 g), the method of Frungillo et al.^20^ was adopted that estimates the level of in vitro NR (EC 1.6.6.1) activity as NAD(P)H-dependent rate of NO_2_^−^ production and was expressed as µmol NO_2_^−^ formed min^−1^ g^−1^ FW. For determination of NiR (EC 1.7.7.1) activity, the reaction mixture contained 0.2 ml NADH (2 mg/ml), 0.6 ml 0.1 M KNO_2_, 0.2 ml enzyme extract and 1 ml Griess reagent (1% sulfanilamide and 0.02% ethylene diamine dihydrochloride) in a total volume of 2 ml. Activity was expressed in µmol NO_2_^−^ reduced min^−1^ g^−1^ FW. The method of Nagy et al.^91^, with some modifications was used to analyze the total GS (EC 6.3.1.2) activity. The method of Singh and Srivastava^92,93^, with some modifications was used to measure the NADH-GOGAT (EC 1.4.1.14), and aminating GDH (NADPH-GDH, EC 1.4.1.4) and deaminating GDH (NADH-GDH, EC 1.4.1.2) activities. The activities were expressed in term of μmol min^−1^ g^−1^ FW. The detailed methodology is given in the supporting file for materials and methods.

#### Activities of key enzymes of NO-metabolism

Roots (100 mg) enzyme extract was prepared in 2 ml of 100 mM HEPES–KOH buffer (pH 7.5) containing 1 M EDTA, 10% glycerol (v/v), 5 M DTT, 0.5 M PMSF, 0.1% Triton X-100 (v/v), 1% polyvinylpyrrolidone (PVP) and 20 µM flavin adinine dinucleotide (FAD) and used for determination of NOSLE activity, according to the method given by Gonzalez et al.^94^, with some modifications. The method of Barraso et al.^95^, with some modifications was adopted to determine the GSNOR (EC 1.2.1.1) activity. This is based on the rate of NADH oxidation in presence of GSNO. The NOSLE and GSNOR activities were calculated using extinction coefficient (ε = 6.22 mM^−1^ cm^−1^) and expressed in nmol min^−1^ mg^−1^ FW. The detailed methodology is given in the supporting file for materials and methods.

#### Protein estimation

Protein was measured following the Bradford^96^ method using bovine serum albumin (BSA) as standard, as described in our previous report^29^.

### RNA extraction, cDNA preparation and gene expression analysis

The expression profiles of *Brassica* genes related to N-uptake and its assimilation such as *BjNRT1.1*, *BjNRT1.2*, *BjNRT1.3*, *BjNRT1.7*, *BjNRT1.8*, *BjNRT2.1*, *BjNRT2.7*, *BjAMT1.1*, *BjAMT1.2*, *BjAMT2*, *BjNR1*, *BjNR2*, *BjNiR*, *BjGS1.1*, *BjGS1.3*, *BjGS2*, *BjFd-GOGAT*, *BjNADH-GOGAT*, *BjGDH1* and *BjGDH* were analyzed. All gene-specific primers and their accession details which were used in this study are listed in supplementary materials (Tables S6, S7). The genes selected in the present work are based on their putative involvement in N uptake and assimilation in *B. juncea* under multiple abiotic stresses^69,97^. Goel and Singh^69^ have been determined the phylogenetic relationship of *B. juncea* proteins with that of their *A. thaliana* orthologs which were found to be clustered together. Further, we have previously shown that NO regulates N assimilation related candidate genes and their enzymes in *B. juncea* under arsenic stress^98^. Therefore, the present study was carried out to examine the changes in similar genes under drought stress.

For total RNA isolation, root tissues were frozen in liquid nitrogen, homogenized in chilled mortar and pestle, and stored immediately at − 80 °C until further use. Total RNA was isolated using RNeasy Plant Mini Kit (Qiagen, cat. no. 74904) according the manufacturer's protocol. The concentration of RNA was quantified by Nano Drop spectrophotometer, and the quality was ascertained on 1.2% agarose gel. Approximately, 2 μg of total RNA was used for first strand cDNA synthesis using RevertAid H Minus First Strand cDNA synthesis kit (Thermo Fisher Scientific, Inc., Waltham, MA, US), as recommended by the manufacturer. Prior to perform real time PCR analysis, isolated RNA was treated with the RNase-free DNase I (Qiagen, cat. no. 79254), and total 20 μl volume was used in a Qiagen RotorGeneQ High Resolution Melt Instrument (Qiagen, cat. no. 9001580) using a SensiFAST SYBR No-ROX One-Step Kit (Bioline, USA, cat. no. BIO-72001). The transcript data was normalized relative to control using *actin* gene of *Brassica* as a reference gene which was found to be stably expressed in different treatment conditions (Supplementary Materials Table S8). The relative gene expression was calculated according to the Comparative Cycle Threshold Method using 2^−ΔΔCT^ formula of Livak and Schmittgen^99^ and expressed as the fold change with 95% confidence interval (CI) (Fig. 6, Supplementary materials Table S5). For each sample, four biological replicates with three technical replicates were assayed under identical conditions.

### Statistical analysis

The one-way analysis of variance (ANOVA) test was performed to analyze the data according CRBD model using SAS (version 9.3, SAS Institute Inc., Cary, NC, USA). The *p*-values for all parameters are given in Supplementary Materials Tables S1 and S3. The significant difference in treatments other than control was represented by asterisk (*)/small letters, above the mean value following the Duncan’s multiple range test (DMRT) or Tukey’s test at a probability level of confidence (*p* ≤ 0.05). The resulted data are mean ± standard error (SE)/ confidence interval (CI) of four biological replicates (n = 4) taken from three independent experiments. The graphs are plotted using scientific software ORIGIN9.1.

## Supplementary Information

Supplementary Information 1.

Supplementary Information 2.
