# Supplementary material for: Root NRT, NiR, AMT, GS, GOGAT and GDH expression levels reveal NO and ABA mediated drought tolerance in Brassica juncea L
Source: Sci Rep. 2021 Apr 12;11:7992. doi: 10.1038/s41598-021-86401-0 (PMC8041993; doi:10.1038/s41598-021-86401-0)
Supplement: Supplementary file 1 — Supplementary Information 1. [file 41598_2021_86401_MOESM1_ESM.pdf]

Supporting file for materials and methods

Root *NRT, NiR, AMT, GS, GOGAT* and *GDH* expression levels reveal NO and ABA mediated drought tolerance in *Brassica juncea* L.

Seema Sahay[1,2,3], Luis Robledo-Arratia[2], Katarzyna Glowacka[3], Meetu Gupta[1]*

[1]Ecotoxicogenomics Lab, Department of Biotechnology, Jamia Millia Islamia, New Delhi-110025, India

[2]Departmento de Biología, Instituto Nacional de Investigaciones Nucleares (ININ), Ocoyoacac, C.P. 52750, México

[3]Department of Biochemistry and Center for Plant Science Innovation, University of Nebraska-Lincoln, Lincoln, NE-68588, USA

*Corresponding author email: meetu_gpt@yahoo.com; mgupta@jmi.ac.in (Meetu Gupta)

Detail methodology

Biochemical analysis

Analysis of total S-nitrosothiol (SNO) and S-nitrosoglutathione (GSNO) content

Root tissues (0.2g) were homogenized in 2ml of extraction solution containing 0.1M phosphate buffer (pH 7.2), 0.1M EDTA and 0.1M EGTA, using the method of Frungillo et al.[79], with some modifications. The homogenized extract was incubated for 5-10 min in equal volume of 1% sulfanilamide dissolved in 0.5M HCl without (solution A) or with 0.2% $HgCl_2$ (solution B) for the development of diazonium salt. Both incubated solutions were added separately with an equal volume of 0.02% N-NED in 0.5M HCl to initiate the reaction, and to obtain the formation of azo dye by incubating for 10 min. The absorbance was measured at 550nm and the SNO content was quantified as difference of absorbance between solution B and A, based upon a hydrolysis of S-nitrosylation in the presence of $HgCl_2$. The values obtained were compared with a standard curve constructed using GSNO.

For GSNO content, root tissues (100mg) were homogenized in 1ml of 0.1M HCl, centrifuged at 15,000 g for 20 min at 4°C. The content of GSNO was measured adopting the liquid chromatography-electrospray mass spectrometry (LC-ES-MS) method of Airaki et al.[80], with some modifications. The supernatant was filtered through 0.22μm polyvinylidene fluoride filter, made photo-protective to avoid the possible degradation of GSNO and other glutathione analytes (such as GSH and GSSG). This process was immediately carried out for detection and quantification of GSNO using LC-ES-MS system of a Waters Allience 2695 HPLC system and a Micromass Quattro API triple quadrupole mass spectrometer. An Atlantis® T33μm 2.1×100 mm column was used in a HPLC system.

Analysis of NO_3^-, NO_2^-, NH_4^+ and total N content

For estimation of NO_3^- content, fresh roots (0.025g) were homogenised in 1.5ml of 20mM HEPES buffer (pH 8.0) using nitro-salicylic acid method of Cataldo et al.[81], with some modifications. The homogenate was centrifuged at 10,000 g for 10 min at 4°C. Supernatant (20μl) was mixed with 5% salicyclic acid (w/v) in concentrated H_2SO_4, and then neutralized by slowly added 2N NaOH to a final volume of 2ml. Solution was cooled at room temperature and the absorbance was measured at 410nm. The solution without salicyclic acid was used as blank. Standard curve was constructed using KNO_3. Subsequently, $NO_2^-$was estimated from the same supernatant as described by Snell and Snell[82]. Equal volume of supernatant was mixed with equal volume of 1% sulphanilamide and 0.02% NED-HCl, absorbance was monitored at 540nm after 15 min incubation. NO_2^-content was calculated against the calibration curve using KNO_2 as standard. For NH_4^+ content, supernatant (100μl) was mixed with 10% K-Na tartrate, distilled water and Nesseler's reagent, according to the method of Molins-Legue et al.[83]. The absorbance was recorded after 5 min incubation at 425nm. NH_4^+ content was calculated against calibration curve using NH_4Cl as standard. Total N content was measured by acid-peroxide digested materials using the method of Lindner's[84]. Oven dried roots (0.1g) were digested with 2ml of concentrated H_2SO_4 by heated in digestion assembly at 80°C for about 2 h. The digested mixture was cooled down and 0.5ml of chemically pure 30% H_2O_2 was added drop by drop, followed by several times heating and cooling to make digested solution colorless. The digested solution was diluted with distilled water to the final volume 100ml. 10 ml of digested solution was mixed with 2.5N NaOH and 10% Na_2SiO_3 was added to neutralize excess of acid and to prevent turbidity, respectively and then distilled water was added to the final volume of 50ml. From this, 5ml aliquot was added with 0.5ml of Nessler's reagent and incubated for 5 min for maximum color

development, and then absorbance was recorded at 520nm. The N content was calculated against standard curve of $(NH_4)_2SO_4$.

Estimation of total free amino acids, proline and proline metabolism enzymes

Ninhydrin method was used to measure total free amino acids, as described by Yokoyama and Hiramatsu's[87], with some modifications. Frozen roots (0.2g) were extracted with 5-10ml 80% ethanol, filtered two times using Whatmann 1 filter paper. Equal volume of filtrate extract was mixed with equal volume of 80% ethanol, L-leucine and 1 ml of ninhydrin (prepared in methyl cellosolve (2 methoxy ethanol)) to a final volume 2ml using distilled water. The mixture was kept on water bath for 20 min. After that 5ml diluents solvent (equal volume of water and n-propanol) was added, incubated at room temperature for 15 min. Developed purplish color was read at 570nm against 80% ethanol which served as blank. L-leucine (Sigma, cat. no. L8000) was used as a standard.

Bates et al.[88] method was used to measure proline content. The detailed methodology has been described in our previous report[27]. The activity of Δ^1-pyrroline-5-carboxylate synthetase (P5CS; EC 2.7.2.11) and proline dehydrogenase (ProDH; EC 1.5.99.8) was estimated according to the method described by Garcia-Rios et al.[89] and Reno and Splittstoesser[90] respectively, with some modifications. For estimating proline metabolism enzymes activities, roots (0.2g) were homogenized with 2ml extraction buffer (containing 100mM Tris-HCl buffer (pH 7.4), 100mM β-mercaptoethanol, 10mM $MgCl_2$ and 1m MPMSF), centrifuged at 10,000 g for 15 min, and enzyme extract was used for the determination of both enzyme activities. For P5CS activity, a reaction mixture (total volume 3ml) contained 100mM Tris-HCl buffer (pH 7.2), 25mM $MgCl_2$, 75mM sodium glutamate, 5mM ATP, 0.4mM NADPH (added in last to initiate the reaction) and 0.2 ml enzyme extract. A decrease in absorbance was recorded for a total 1 min at 30 sec interval to measure the activity, as the rate of NADPH consumption at 340nm. Reaction mixture (total volume 3ml) for ProDH activity contained 100mM $Na_2CO_3^-$-$NaHCO_3$ (pH 10.3), 20mM L-Pro, 10mM NAD^+ (added in last to initiate the reaction) and 0.5ml of enzyme extract. The increase in an absorbance was measured at 340nm.

Activities of key enzymes of N-metabolism

In-vitro NR (EC 1.6.6.1**)** activity was estimated as NAD(P)H-dependent rate of NO_2^- production as previously described by Frungillo et al.[20] with some modifications. Briefly,

0.2g fresh roots were homogenized in 2ml extraction buffer containing 0.1M HEPES-KOH buffer (pH 7.5), 3% polyvinylpolypyrrolidone (PVP w/v), 1mM EDTA and 7mM cysteine, centrifuged at 12,000 g for 15 min at 4°C. The reaction mixture of 50mM HEPES-KOH (pH 7.5), 100mM NADH (added in last to initiate the reaction), 5mM KNO_3, 6mM $MgCl_2$ and 100µl enzymatic extract was incubated for 30 min at 25°C. Activation in NR activity *in-vitro* was measured by determining the NO_2^- formation after adding the equal volume of 1% sulfanilamide (w/v) and 0.02% (N-NED; w/v) in 1.5 N HCl, followed by 5 min incubation for maximum colour development. Reaction was terminated by adding 50µl Zn-acetate and the absorbance was measured at 540nm. The reaction mixture without NADH was used as the blank. The KNO_2 was used as a standard.

For determination of NiR (EC 1.7.7.1) activity, roots (100 mg) were homogenized in 2ml of 0.1M phosphate buffer (pH=7.5) containing cysteine and EDTA followed by centrifugation for 15 min at 4000 g. The reaction mixture contained 0.2ml NADH (2mg/ml), 0.6ml 0.1 M KNO_2, 0.2ml homogenate (enzyme extract) and 1ml Griess reagent (1% sulfanilamide and 0.02% ethylene diamine dihydrochloride) in a total volume of 2ml. The Griess reagent was added in the last to the reaction mixture to stop the reaction, followed by centrifugation for 5min at 4000 g. The absorbance was read at 540nm using KNO_2 as a standard.

Roots (0.2g) were homogenized in 2 ml of extraction buffer (containing 1mM EDTA, 50mM Tris buffer, 10% glycerol (v/v), and 5mM 2-merceptoethonal, pH 8.0), followed by centrifugation at 14,000 g for 5 min. The enzyme extract was used for total GS (EC 6.3.1.2) activity, adopting the method of Nagy et al.[91], with some modifications. The reaction mixture (2ml) contained 50mM imidzole, 30mM $MgCl_2$, 25mM hydroxylamine and 100mM Na-glutamate (pH 7.0) and enzyme extract (1 ml) was incubated at 37°C for 30-40 min. Reaction was terminated by adding 0.8ml acidic $FeCl_3$ solution (24% TCA, w/v and 10% $FeCl_3$ w/v in 18% HCl). The formation of γ-glutamyl monohydroxamate was measured spectrophotometrically at 540nm.

The method of Singh and Srivastava[92,93], with some modifications was used to measure the GOGAT and GDH activity. For NADH-GOGAT (EC 1.4.1.14) activity, root tissues (0.2g) were homogenized with 2ml 0.2M Na-phosphate buffer (pH 7.5) containing 2mM EDTA, 50mM KCl, 0.1% (*v*/*v*) mercaptoethanol and 0.5% (*v*/*v*) TritonX-100, followed by centrifugation (10,000 g, 4°C, 15 min). Enzyme extract (0.3ml) was mixed with 20mM L-

glutamine (added in last to initiate the reaction), 0.1M 2-oxoglutarate, 10mM KCI, and 3mM NADH in a final volume of 1ml. The decrease in absorbance was measured at 340nm for 3 min. For aminating GDH (NADPH-GDH, EC 1.4.1.4) and deaminating GDH (NADH-GDH, EC 1.4.1.2), fresh roots (0.2g) were homogenised with 2ml 0.5M Na-phosphate buffer, pH 7.4 (containing 0.4M sucrose and 2mM EDTA) using ice-cold mortar and pestle, followed by centrifugation at 15,000 g for 15 min at 4°C. The reaction mixture (1ml) contained 0.1M sodium phosphate buffer (pH 8.1), 0.2M 2-oxoglutarate, 1.5M ammonium sulphate substrate (added in last to initiate the reaction), 1mM NADPH (NADH in case of NADH-GDH) and 0.2ml of enzyme extract. Decrease in absorbance was monitored at 340nm for total 3 min. NADPH/NADH was used as a calibration standard to calculate GDH enzyme activity.

Activities of key enzymes of NO-metabolism

The NOSLE activity was determined according to the method as given by Gonzalez et al.[94], with some modifications. Root samples (100mg) were homogenized in 2ml of extraction buffer containing100mM HEPES-KOH buffer (pH 7.5), 1M EDTA, 10% glycerol (v/v), 5M DTT, 0.5M PMSF, 0.1% Triton X-100 (v/v), 1% polyvinylpyrrolidone (PVP) and 20µM flavin adinine dinucleotide (FAD). The homogenate was centrifuged at 13,000 g for 20 min at 4°C. Resultant enzyme extract (200µl) was added with the reaction mixture containing 100mM phosphate buffer (pH 7.0), 1mM L-Arg, 2mM $MgCl_2$, 0.3mM $CaCl_2$, 4µM BH_4, 1µM FAD, 1µM flavin mononucleotide (FMN), 0.2mM DTT, 0.2mM NAD(P)H to a final volume of 2ml. Consumption of NAD(P)H was monitored as a decrease in an absorbance at 340nm for total 5min.

The method of Barraso et al.[95], with some modifications was adopted to determine the GSNOR (EC 1.2.1.1) activity. This is based on the rate of NADH oxidation in presence of GSNO. Briefly, root extract (100µl) was incubated with reaction mixture containing 20mM Tris HCl buffer(pH 8.0), 0.5mM EDTA, 350µM NADH and 350µM GSNO (added in last to terminate the reaction) to a final volume of 1 ml. Activity was measured as an absorbance difference between NADH oxidation in the absence and presence of GSNO at 340nm.
