# Supplementary material for: Root NRT, NiR, AMT, GS, GOGAT and GDH expression levels reveal NO and ABA mediated drought tolerance in Brassica juncea L
Source: Sci Rep. 2021 Apr 12;11:7992. doi: 10.1038/s41598-021-86401-0 (PMC8041993; doi:10.1038/s41598-021-86401-0)
Supplement: Supplementary file 2 — Supplementary Information 2. [file 41598_2021_86401_MOESM2_ESM.pdf]

Supplementary materials

Root *NRT*, *NiR*, *AMT*, *GS*, *GOGAT* and *GDH* expression levels reveal NO and ABA mediated drought tolerance in *Brassica juncea* L.

Seema Sahay[1,2,3], Luis Robledo-Arratia[2], Katarzyna Glowacka[3], Meetu Gupta[1]*

[1]Ecotoxicogenomics Lab, Department of Biotechnology, Jamia Millia Islamia, New Delhi-110025, India

[2]Departmento de Biología, Instituto Nacional de Investigaciones Nucleares (ININ), Ocoyoacac, C.P. 52750, México

[3]Department of Biochemistry and Center for Plant Science Innovation, University of Nebraska-Lincoln, Lincoln, NE-68588, USA

*Corresponding author email: meetu_gpt@yahoo.com; mgupta@jmi.ac.in (Meetu Gupta)

This file includes:

Tables S1 to S8

Supplementary Table S1 One way ANOVA *$p>0.05$* values indicate no significant difference between control and NO or ABA treatments for different studied parameters.

**Parameters**	***p*-values (NO)**	***p*-values (ABA)**	**Parameters**	***p*-values (NO)**	***p*-values (ABA)**
Shoot length	0.476	0.593	S	0.596	0.999
Root length	0.066	0.136	N	0.999	1
Fresh weight	0.0048	0.148	NO_3^-	0.828	0.993
Dry weight	0.136	0.963	NO_2^-	0.792	1
Total free amino acids	0.998	1	NH_4^+	0.972	0.420
Proline	1	1	NH_4^+/ NO_2^-	1	0.969
P5CS	0.974	0.943	NR	0.999	0.992
ProDH	0.999	1	NiR	0.986	0.999
Fe	0.381	1	GS	0.798	1
Cu	0.183	1	GOGAT	0.999	1
Mn	0.106	0.914	NADPH-GDH	0.703	0.964
Zn	0.236	1	NADH-GDH	0.693	0.952
P	0.804	0.820	SNO	0.0004	0.0019
K	0.992	1	GSNO	0.0062	0.311
Ca	0.401	1	NOSLE	0.907	1
Mg	0.219	1	Evans blue uptake	0.911	0.946
Na	0.901	0.426	GSNOR	0.071	0.490

Supplementary Table S2 Percent (%)-increase or decrease in all growth, biochemical and physiological parameters of 7-d-old *B. juncea* seedlings treated for another 96 h with PEG, NO, ABA and their combinations (nitrate agonists). (i) All treatments were compared with control. (ii) Nitrate agonist treatments were compared with PEG alone.

**Parameters**	**%-increase or decrease**								
	**(i) All treatments vs. Control**						**(ii) PEG vs. Nitrate agonist treatments**		
	**NO**	**ABA**	**PEG**	**PEG+NO**	**PEG+NO +ABA**	**PEG+ABA**	**PEG+NO**	**PEG+NO +ABA**	**PEG+AB A**
Evans Blue uptake	17.5	14.1	193.5	52.5	61.5	71.7	48.0	43.9	39.4
Total free amino acids	7.2	1.4	84.0	18.8	27.5	39.1	35.4	30.7	24.4
Proline	-3.6	-2.4	37.3	21.8	9.7	31.6	-11.2	-20.1	-4.2
P5CS	-2.9	-4.2	60.5	15.7	30.2	-35.1	-27.8	-18.8	-15.7
ProDH	2.1	0.41	-28.6	-6.8	-11.6	-18.0	30.5	23.8	14.9
Total SNO	20.4	17.6	28.3	38.5	32.1	35.6	7.8	2.9	5.6
GSNO	50.0	30.6	62.9	96.7	90.3	82.2	20.7	16.8	11.8
NOSLE	12.0	5.0	27.0	52.0	44.0	40.0	19.6	13.3	10.2
GSNOR	6.4	4.7	-30.6	-9.9	-17.5	-22.4	29.7	18.8	11.7
NO_3^-	10.5	-2.6	-52.6	-13.1	-21.0	34.2	83.3	66.5	38.4
NO_2^-	13.6	4.5	-59.0	-18.1	-22.7	-36.3	98.9	88.2	55.1
NH_4^+	14.2	28.5	125.0	71.4	82.1	96.3	-23.8	-19.0	-12.6
NH_4^+/NO_3^-	0.78	22.8	451.9	109.4	137.0	208.6	-62.0	-57.0	-44.0
N	3.3	-2.3	-33.3	-8.7	-14.7	-20.0	36.9	27.8	19.9
NR	3.2	-4.5	-39.2	-13.0	-16.9	-24.1	43.0	36.5	24.7
NiR	6.2	-3.9	-48.0	-18.1	-25.1	-32.2	57.5	43.9	30.3

Continued from previous page

Parameters	%-increase or decrease								
	(i) All treatments vs. Control						(ii) PEG vs. Nitrate agonist treatments		
	NO	ABA	PEG	PEG+NO	PEG+NO +ABA	PEG+ABA	PEG+NO	PEG+NO +ABA	PEG+AB A
GS	10.4	1.3	-39.7	-10.2	-15.2	-23.7	48.8	40.6	26.4
NADH-GOGAT	8.6	6.7	-38.4	-7.5	-15.1	-26.4	50.6	38.3	19.8
NADPH-GDH	1.6	-1.3	55.2	8.1	15.3	29.4	-30.3	-25.6	-16.6
NADH-GDH	23.5	16.0	142.4	56.6	62.2	89.6	-35.4	-33.0	-21.7
P	18.2	17.6	-46.5	-2.5	-9.5	-17.4	82.2	69.2	54.5
K	7.4	1.1	-54.4	-1.8	-2.1	-24.4	115.3	114.5	65.6
Ca	25.2	-1.8	27.0	-2.2	-3.2	-7.7	33.9	32.7	26.4
Mg	24.2	2.7	-44.7	-2.1	-10.8	-23.3	77.0	61.3	38.6
Na	-7.2	-13.3	-62.9	-9.1	-14.6	-24.1	145.0	30.2	104.8
S	15.2	4.0	-29.6	-3.3	-7.8	-11.9	37.3	30.9	25.1
Fe	27.3	2.9	-39.9	31.0	-15.1	-16.3	118.1	41.2	39.1
Cu	33.0	1.6	-21.4	-4.9	8.2	9.0	9.5	5.7	4.7
Mn	23.6	17.8	-36.4	13.4	-16.7	16.3	35.3	30.1	30.7
Zn	28.6	-0.60	-38.4	-4.8	-7.3	16.4	54.4	50.4	35.6

Negative values represent % decrease, whereas positive values represent % increase.

Supplementary Table S3 One way ANOVA *$p<0.05$* values for different studied parameters.

**Parameters**	***p*-values**	**Parameters**	***p*-values**	**Parameters**	***p*-values**
Shoot length	<.0001	N	0.0022	*NRT1.3*	<.0001
Root length	<.0001	NO_3^-	<.0001	*NRT1.7*	<.0001
Fresh weight	<.0001	NO_2^-	<.0001	*NRT1.8*	<.0001
Dry weight	<.0001	NH_4^+	<.0001	*NRT2.1*	<.0001
Total free amino acids	0.0002	NH_4^+/ NO_2^-	<.0001	*NRT2.7*	<.0001
Proline	0.0484	NR	<.0001	*AMT1.1*	<.0001
P5CS	<.0001	NiR	<.0001	*AMT1.2*	<.0001
ProDH	0.0003	GS	<.0001	*AMT2*	<.0001
Fe	0.0003	GOGAT	0.032	*NR1*	<.0001
Cu	0.0165	NADPH-GDH	0.0373	*NR2*	<.0001
Mn	<.0001	NADH-GDH	<.0001	*NiR*	<.0001
Zn	<.0001	SNO	0.0025	*GS1.1*	<.0001
P	0.0011	GSNO	<.0001	*GS1.3*	<.0001
K	<.0001	NOSLE	0.0015	*GS2*	<.0001
Ca	0.0213	Evans blue uptake	<.0001	*Fd-GOGAT*	<.0001
Mg	<.0001	GSNOR	0.0154	*NADH-GOGAT*	<.0001
Na	<.0001	*NTR1.1*	<.0001	*GDH1*	<.0001
S	0.0021	*NRT1.2*	<.0001	*GDH2*	<.0001

Supplementary Table S4 The macro- and micro-nutrients contents of 7-d-old *B. juncea* seedlings treated for another 96 h with PEG, NO, ABA and their combinations (nitrate agonists). Values are mean ± S.E. of three independent experiments, each including four biological replicates. Different letters represent significant difference between treatments, where “a” corresponds to the highest value and “d” or “e” to the lowest value.

**Treatments**	**Macronutrients**					
	**P**	**K**	**Ca**	**Mg**	**Na**	**S**
**Control**	12.05±1.0^{ab}	6.01±0.72^{ab}	2.18±0.22^{ab}	10.62±1.0^{ab}	15.86±1.1^{a}	14.65±0.83^{bc}
**NO**	14.25±1.7^{a}	6.46±0.35^{a}	2.73±0.31^{a}	13.2±0.70^{a}	14.55±0.60^{ab}	16.88±0.69^{a}
**ABA**	14.18±1.0^{a}	6.08±0.27^{ab}	2.14±0.13^{ab}	10.91±1.1^{ab}	13.58±0.86^{bc}	15.25±0.66^{ab}
**PEG**	6.44±0.63^{d}	2.74±0.37^{e}	1.59±0.11^{e}	5.87±0.44^{e}	5.81±51^{e}	10.31±0.65^{e}
**PEG+NO**	11.74±1.1^{b}	5.90±0.56^{bc}	2.13±0.18^{bc}	10.39±0.74^{b}	14.24±0.82^{ab}	14.16±0.77^{bc}
**PEG+NO+ABA**	10.90±1.0^{bc}	5.88±0.30^{bc}	2.11±0.15^{bc}	9.47±0.26^{bc}	13.38±0.67^{bc}	13.5±0.96^{cd}
**PEG+ABA**	9.95±0.77^{c}	4.54±0.32^{d}	2.01±0.07^{d}	8.14±0.40^{d}	11.9±0.74^{d}	12.9+0.97^{d}

	**Micronutrients**			
	**Fe**	**Cu**	**Mn**	**Zn**
**Control**	0.238±0.021^{ab}	0.012±0.001^{ab}	0.076±0.004^{ab}	0.164±0.008^{b}
**NO**	0.303±0.023^{a}	0.016±0.002^{a}	0.094±0.005^{a}	0.211±0.011^{a}
**ABA**	0.245±0.028^{ab}	0.012±0.001^{ab}	0.089±0.002^{a}	0.163±0.010^{b}
**PEG**	0.143±0.013^{d}	0.0090±0.001^{d}	0.048±0.005^{d}	0.101±0.009^{e}
**PEG+NO**	0.312±0.029^{a}	0.012±0.001^{ab}	0.066±0.004^{bc}	0.156±0.007^{bc}
**PEG+NO+ABA**	0.202±0.025^{bc}	0.011±0.00^{bc}	0.063±0.001^{bc}	0.152±0.014^{bc}
**PEG+ABA**	0.199±0.011^{bc}	0.011±0.001^{bc}	0.063±0.002^{bc}	0.137±0.005^{d}

Supplementary Table S5 Relative expression levels (fold change values) of N uptake, transport and utilization representative key genes of 7-d-old *B. juncea* seedlings treated for another 96 h with PEG, NO, ABA and their combinations. Values are mean of four biological replicates taken from three independent experiments. Values in parentheses show 95% CI with upper and lower bounds. Same lowercase letters (a-e) represent no significant difference between treatments, determined by Tukey's test.

**Genes**	**NO**	**ABA**	**PEG**	**PEG+NO**	**PEG+NO+ABA**	**PEG+ABA**
NRT1.1	1.40 (1.31, 1.49)^{c}	1.23 (1.15, 1.31)^{c}	0.62 (0.53, 0.71)^{e}	3.25 (2.96, 3.54)^{a}	2.80 (2.44, 3.17)^{a}	2.01 (1.90, 2.12)^{b}
NRT1.2	1.31 (1.24, 1.37)^{c}	1.22 (1.15, 1.29)^{c}	0.73 (0.66, 0.80)^{d}	3.80 (3.19, 4.41)^{a}	3.21 (2.71, 3.73)^{ab}	2.49 (2.25, 2.74)^{b}
NRT1.3	1.23 (1.17, 1.28)^{d}	1.09 (1.05, 1.13)^{d}	0.53 (0.45, 0.62)^{e}	2.52 (2.07, 2.98)^{a}	2.16 (1.96, 2.38)^{ab}	1.74 (1.58, 1.92)^{c}
NRT1.7	1.22 (1.18, 1.28)^{d}	1.01 (0.88, 1.06)^{d}	2.61 (2.42, 2.88)^{a}	1.71 (1.45, 1.97)^{c}	1.89 (1.59, 2.19)^{bc}	2.19 (1.91, 2.49)^{b}
NRT1.8	1.20 (1.16, 1.25)^{c}	1.19 (1.11, 1.28)^{c}	0.44 (0.36, 0.51)^{d}	1.76 (1.55, 1.97)^{a}	1.51 (1.33, 1.71)^{ab}	1.38 (1.27, 1.50)^{b}
NRT2.1	1.30 (1.24, 1.35)^{c}	1.09 (1.05, 1.15)^{c}	0.72 (0.65, 0.79)^{d}	2.89 (2.37, 3.42)^{a}	2.61(2.25, 2.97)^{a}	2.08 (2.06, 2.11)^{b}
NRT2.7	1.51 (1.45, 1.57)^{c}	1.19 (1.14, 1.26)^{c}	0.39 (0.33, 0.46)^{d}	2.32 (2.03, 2.62)^{a}	1.79 (1.62, 1.97)^{b}	1.81 (1.64, 1.98)^{b}
AMT1.1	1.11 (1.06, 1.15)^{c}	1.10 (0.99, 1.22)^{c}	0.22 (0.02, 0.42)^{d}	2.29 (1.92, 2.67)^{a}	1.99 (1.68, 2.32)^{ab}	1.71 (1.51, 1.91)^{b}
AMT1.2	0.99 (0.88, 1.09)^{d}	1.01 (0.86, 1.05)^{d}	4.45 (4.02, 4.90)^{a}	2.80 (2.52, 3.08)^{c}	2.80 (2.77, 2.83)^{c}	3.39 (2.85, 3.94)^{bc}
AMT2	1.20 (1.15, 1.25)^{c}	1.10 (1.02, 1.18)^{d}	0.17 (0.08, 0.26)^{dc}	3.30 (2.84, 3.76)^{a}	2.62 (2.26, 2.98)^{a}	1.82 (1.58, 2.08)^{b}
NR1	1.31(1.24, 1.37)^{c}	1.20 (1.09, 1.31)^{c}	0.70 (0.62, 0.79)^{d}	1.90 (1.62, 2.18)^{a}	1.52 (1.22, 1.83)^{ab}	1.49 (1.16, 1.39)^{b}
NR2	1.39 (1.32, 1.47)^{c}	1.01 (0.88, 1.08)^{d}	0.52 (0.45, 0.59)^{e}	1.86 (1.56, 2.18)^{a}	1.55(1.24, 1.86)^{ab}	1.31 (1.16, 1.46)^{b}
NiR	1.40 (1.32, 1.47)^{b}	1.01 (0.88, 1.08)^{c}	0.40 (0.31, 0.49)^{d}	1.86 (1.62, 2.12)^{a}	1.80 (1.59, 1.01)^{a}	0.98 (0.92, 1.06)^{c}
GS1.1	1.21 (1.17, 1.25)^{bc}	1.16 (1.08, 1.23)^{d}	0.21 (0.11, 0.31)^{e}	1.56 (1.32, 1.80)^{a}	1.29 (1.15, 1.44)^{ab}	1.15 (1.06, 1.24)^{cd}
GS1.3	1.11 (1.03, 1.18)^{c}	1.11 (1.04, 1.18)^{c}	0.63 (0.53, 0.67)^{d}	1.88 (1.63, 2.14)^{a}	1.73 (1.54, 1.92)^{a}	1.21 (1.11, 1.33)^{b}
GS2	1.70 (1.69, 1.91)^{a}	1.30 (1.20, 1.40)^{b}	0.84 (0.77, 0.91)^{c}	1.61 (1.39, 1.83)^{a}	1.33 (1.17, 1.49)^{ab}	1.29 (1.17, 1.42)^{b}
Fd-GOGAT	1.29 (1.23, 1.36)^{c}	1.19 (1.14, 1.26)^{c}	0.52 (0.28, 0.76)^{d}	2.66 (2.38, 2.94)^{a}	2.59 (2.32, 2.88)^{a}	1.99 (1.74, 2.25)^{b}
NADH-GOGAT	1.11 (0.99, 1.22)^{d}	1.11 (1.06, 1.15)^{d}	0.80 (0.71, 0.90)^{e}	3.73 (3.42, 4.02)^{a}	3.11 (2.90, 3.34)^{bc}	2.60 (2.32, 2.90)^{c}
GDH1	1.40 (1.31, 1.48)^{d}	1.30 (1.23, 1.37)^{d}	3.84 (3.29, 4.40)^{a}	1.82 (1.42, 2.25)^{c}	1.98 (1.79, 2.15)^{c}	2.87 (2.61, 3.11)^{b}
GDH2	1.60 (1.45, 1.75)^{b}	1.21 (1.15, 1.27)^{d}	2.73 (2.53, 2.94)^{a}	1.48 (1.23, 1.60)^{c}	1.41 (1.16, 1.50)^{c}	1.89 (1.69, 2.09)^{b}

Supplementary Table S6 List of 20 primer sequences of nitrogen uptake and assimilation genes of *B. juncea* used in RT-PCR study.

S. No.	Genes	Primer sequence 5'-3'	
1.	*BjNRT1.1*	**F:** CTTCCTCGGAACTTCGTTCATG	**R:** TTGGATAGCGGCGAATATAGC
2.	*BjNRT1.2*	**F:** ATCTGGCGAATGCAAGTAACCT	**R:** GCTGTGCCCATGAAGTTGGT
3.	*BjNRT1.3*	**F:** CAAGCCATGTTTATGGAGCGTAA	**R:** TAAGGGAAGTGAAGAGGAGAATGGT
4.	*BjNRT1.7*	**F:** TCGGCGCGTTTATCTCAGA	**R:** GGTGGCGAATGAGGCAAA
5.	*BjNRT1.8*	**F:** AATGCAGAAGCTGCCAATAGTG	**R:** TGTAGCGTCCCCAATAAGAGTCA
6.	*BjNRT2.1*	**F:** AAGAACATGCTTCCCCACTGA	**R:** TCACAACCGAACAAGGGCTAA
7.	*BjNRT2.7*	**F:** TCCTAGCGCTTCTGTACGGTTAC	**R:** GCCTCTAGATTCACGCCGAAT
8.	*BjAMT1.1*	**F:** CAAACGGCTTCATCGGAAAA	**R:** GCGATAGCAAAAGCCCATTG
9.	*BjAMT1.2*	**F:** CCACAGCTTTTTCGCCTTGA	**R:** GATTCCCGCTGCGGCTATA
10.	*BjAMT2*	**F:** CAGTTCCATGGGCCTCTATGA	**R:** CCGGCTACTGCGTGTGTGTA
11.	*BjNR1*	**F:** TCCGGCGAAGTTCACCAT	**R:** CGGCGCATACGAGAGTCA
12.	*BjNR2*	**F:** GTTTACTTCAAGAACGTCCATCCA	**R:** CGGTAGCGAGTCCAAATGCT
13.	*BjNiR*	**F:** CCAAAAGATGCGAAGAGGCTAT	**R:** CTTTGCAGAGTGGAAGAACATCA
14.	*BjGS1.1*	**F:** GCCTCTTGGTTGGCCTATTG	**R:** AGCATCAACGATGTCTCTACCAAA
15.	*BjGS1.3*	**F:** CGACTCCACCAAGCAAATCA	**R:** ATCGCTCACTGGTCCTGGAA
16.	*BjGS2*	**F:** CACATCAAATCCTCCTTCCTCTCT	**R:** AACACTTGATCCCAAACCCATAGA
17.	*BjFd-GOGAT*	**F:** GCCTTGCGCGAACTCAA	**R:** GGACGGTCTCTACAGCCAGATATG
18.	*BjNADH-GOGAT*	**F:** GCCTTGCGCGAACTCAA	**R:** CCAGATATGGGCAGATAGCATCT
19.	*BjGDH1*	**F:** AACCCCTGGATGACAAAACG	**R:** GAGACGCGGCTACAGGAAGA
20.	*BjGDH2*	**F:** CCTGAAGGCATCGACATCAA	**R:** GCAGCTCCTCAGAGTCCATAGC
21.	*BjACT*	**F:** TTCAATGTCCCTGCCATGTA	**R:** TTGATCTTCATGCTGCTTGG

Supplementary Table S7 Accession numbers of nitrogen uptake and assimilation genes of *B. juncea* used in RT-PCR study.

**Genes**	**Accession numbers**	**Description**
BjNRT1.1	KT119578	Low affinity nitrate transporter 1.1
BjNRT1.2	KT119579	Low affinity nitrate transporter 1.2
BjNRT1.3	KT119580	Low affinity nitrate transporter 1.3
BjNRT1.7	KT119583	Low affinity nitrate transporter 1.7
BjNRT1.8	KT119584	Low affinity nitrate transporter 1.8
BjNRT2.1	KT119585	High affinity nitrate transporter 2.1
BjNRT2.7	KT119586	High affinity nitrate transporter 2.7
BjAMT1.1	KT119596	Ammonium transporter 1.1
BjAMT1.2	KT119597	Ammonium transporter 1.2
BjAMT2	KT119598	Ammonium transporter 2
BjNR1	KT119587	Nitrate reductase 1
BjNR2	KT119588	Nitrate reductase 1
BjNiR	KT119589	Nitrite reductase
BjGS1.1	KT119590	Cytoplasmic glutamine synthetase 1.1
BjGS1.3	KT119591	Cytoplasmic glutamine synthetase 1.3
BjGS2	KT119593	Cytoplasmic glutamine synthetase 2
BjFd-GOGAT	KT119603	Ferredoxin-dependent glutamate synthase
BjNADH-GOGAT	KT119604	NADH-dependent glutamate synthase 1
GDH1	KT119594	Chloroplastic glutamate dehydrogenase 1
GDH2	KT119595	Mitochondrial glutamate dehydrogenase 2

Supplementary Table S8 Ct values of *B. juncea* reference gene *actin* showing stable expression at different treatment levels of PEG, NO, ABA and their combinations.

Samples	Replicates	Ct values		
		**Experiment 1**	**Experiment 2**	**Experiment 3**
Control	R1	18.23	17.66	18.45
	R2	19.01	18.12	17.93
	R3	18.74	18.12	18.02
	R4	18.83	18.03	18.63
NO	R1	19.12	18.17	18.15
	R2	18.67	18.16	17.82
	R3	18.99	18.22	17.91
	R4	18.45	18.18	18.38
ABA	R1	17.93	19.11	18.31
	R2	18.02	18.84	18.22
	R3	18.23	18.83	18.63
	R4	18.55	19.22	18.82
PEG	R1	17.78	18.76	18.53
	R2	17.94	18.55	18.11
	R3	18.21	18.45	18.76
	R4	18.12	17.93	18.42
PEG+NO	R1	18.33	17.91	17.85
	R2	18.31	17.77	17.91
	R3	18.16	18.32	18.32
	R4	18.44	18.41	18.47
PEG+ABA+NO	R1	18.32	18.57	18.38
	R2	18.11	19.03	19.13
	R3	18.67	18.66	18.76
	R4	18.24	18.83	18.83
PEG+ABA	R1	17.85	19.12	18.88
	R2	17.91	18.67	18.61
	R3	18.22	18.22	18.18
	R4	18.12	18.44	18.54
